# Therapeutic Delivery of Pip4k2c‐Modified mRNA Attenuates Cardiac Hypertrophy and Fibrosis in the Failing Heart

**DOI:** 10.1002/advs.202004661

**Published:** 2021-03-12

**Authors:** Ajit Magadum, Neha Singh, Ann Anu Kurian, Mohammad Tofael Kabir Sharkar, Nishat Sultana, Elena Chepurko, Keerat Kaur, Magdalena M. Żak, Yoav Hadas, Djamel Lebeche, Susmita Sahoo, Roger Hajjar, Lior Zangi

**Affiliations:** ^1^ Cardiovascular Research Center Icahn School of Medicine at Mount Sinai New York NY 10029 USA; ^2^ Department of Genetics and Genomic Sciences Icahn School of Medicine at Mount Sinai New York NY 10029 USA; ^3^ Black Family Stem Cell Institute Icahn School of Medicine at Mount Sinai New York NY 10029 USA; ^4^ Phospholamban Foundation Amsterdam The Netherlands

**Keywords:** fibrosis, gene therapy, heart failure, hypertrophy

## Abstract

Heart failure (HF) remains a major cause of morbidity and mortality worldwide. One of the risk factors for HF is cardiac hypertrophy (CH), which is frequently accompanied by cardiac fibrosis (CF). CH and CF are controlled by master regulators mTORC1 and TGF‐*β*, respectively. Type‐2‐phosphatidylinositol‐5‐phosphate‐4‐kinase‐gamma (Pip4k2c) is a known mTORC1 regulator. It is shown that Pip4k2c is significantly downregulated in the hearts of CH and HF patients as compared to non‐injured hearts. The role of Pip4k2c in the heart during development and disease is unknown. It is shown that deleting Pip4k2c does not affect normal embryonic cardiac development; however, three weeks after TAC, adult Pip4k2c^−/−^ mice has higher rates of CH, CF, and sudden death than wild‐type mice. In a gain‐of‐function study using a TAC mouse model, Pip4k2c is transiently upregulated using a modified mRNA (modRNA) gene delivery platform, which significantly improve heart function, reverse CH and CF, and lead to increased survival. Mechanistically, it is shown that Pip4k2c inhibits TGF*β*1 via its N‐terminal motif, Pip5k1*α*, phospho‐AKT 1/2/3, and phospho‐Smad3. In sum, loss‐and‐gain‐of‐function studies in a TAC mouse model are used to identify Pip4k2c as a potential therapeutic target for CF, CH, and HF, for which modRNA is a highly translatable gene therapy approach.

## Graphical abstract

Heart failure remains a major cause of morbidity and mortality worldwide. One of the risk factors for HF is cardiac hypertrophy (CH), which is frequently accompanied by cardiac fibrosis (CF). Here, gain‐ and loss‐of‐function studies are used to identify the therapeutic role of Pip4k2c in preventing CH and CF via inhibition of mTOR and TGF*β*1 pathways, respectively.

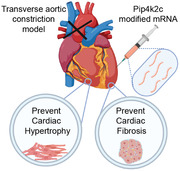

## 1 Introduction

HF is a major worldwide health problem and a huge socioeconomic burden.^[^ ^1^ ^]^ Pathological growth and hypertrophy (CH) develop due to either sustained pressure overload in the injured heart or genetic mutation in the non‐injured heart, resulting in ventricular chamber enlargement and contractile dysfunction often accompanied by an increased immune response, cardiac fibrosis (CF), and scarring that subsequently contribute to HF.^[^ ^2^, ^3^ ^]^ More common causes of CF, which is a key risk factor for arrhythmia and sudden death, are hypertension, valve issues, and congenital heart disease. Unraveling the mechanistic molecular pathways that underlie pathological stress and HF may lead to the development of new therapeutic options for CH and CF in HF.^[^ ^4^, ^5^, ^6^, ^7^, ^8^, ^9^ ^]^

Pip4k2c is a Type 2 phosphatidylinositol‐5‐phosphate 4‐kinase (PI5P4K) that converts phosphatidylinositol‐5‐phosphate to Phosphatidylinositol 4,5‐bisphosphate in mammals. The mammalian gene PI5P4K encodes three enzymes – PI5P4K*α*, PI5P4K*β*, and PI5P4K*γ* – that play important roles in development, homeostasis, and disease.^[^ ^10^, ^11^, ^12^ ^]^ Pip4k2c is expressed primarily in the kidney, brain, heart, and testes.^[^ ^13^, ^14^, ^15^ ^]^ To date, Pip4k2c has been studied for its regulatory roles in insulin resistance, via catalytic‐independent suppression of PIP5k synthesis,^[^ ^16^ ^]^ and immune response, via mTORC1‐signaling inhibition in the kidney, testes, muscle, or brain.^[^ ^10^, ^11^, ^16^ ^]^ The N‐terminal motif of Pip4k2c was found to be crucial to mTOR inhibition.^[^ ^16^ ^]^ Research produced during the last decade has clarified that mTORC1 signaling is important to promoting CH in response to chronic pressure overload.^[^ ^9^, ^17^, ^18^, ^19^, ^20^, ^21^ ^]^ Moreover, TGF*β*1 engenders CF via the downstream mediator Smad3, which activates the extracellular matrix (ECM)‐related genes.^[^ ^22^ ^]^ What remains unclear, however, is 1) the role of pip4k2c in the heart during development and disease, 2) whether or not Pip4k2c affects TGF*β*1 signaling in cardiovascular pathophysiology, and 3) the nature of the interplay between Pip4k2c and mTORC1 in the context of cardiac disease. Here, we use gain‐ and loss‐of‐function studies to explore the role of Pip4k2c in preventing cardiac hypertrophy and fibrosis in the failing heart.

## 2 Results

### 2.1 Pip4k2c Expression Decreases during Human and Mouse Heart Disease

**Figure 1:**
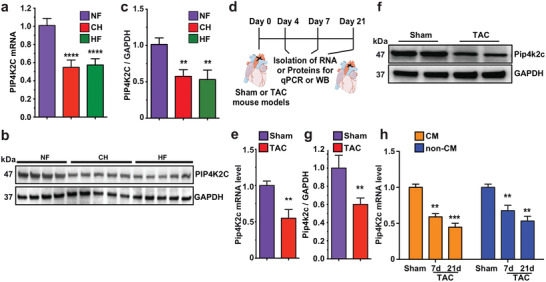
PIP4K2C and Pip4k2c expression decreases in failing hearts of humans and mice, respectively. a) PIP4K2C mRNA expression in human non‐injured normal left ventricle (LV) (non‐failing, NF, *n* = 5), cardiac hypertrophy (CH, *n* = 5), and heart failure (HF, *n* = 5). b. Western blot of PIP4K2C expression in human LV samples with GAPDH as control (*n* = 4 for NF, *n* = 5 for CH, *n* = 5 for HF). c) Quantitative analysis of b. d) Experimental timeline to analyze Pip4k2c expression in sham‐operated or TAC mouse model. e) mRNA expression of Pip4k2c in WT mouse heart samples compared to those from TAC recipients, 4 days after injury (*n* = 4). f) Western blot evaluation of Pip4k2c protein in WT mouse heart samples compared to those from TAC recipients 4 days after injury, with GAPDH as a control housekeeping gene. g) Quantitative analysis of Pip4k2c western blot from f (*n* = 2). h) Pip4k2c mRNA expression in CM or non‐CMs after Sham or 7 or 21 days post TAC injury (*n* = 3). One‐way ANOVA, Tukey's Multiple Comparison Test were used in a,c; unpaired two‐tailed *t*‐test was used in e,g; and two‐way ANOVA and Bonferroni post‐hoc tests were used in h. ****, *P* < 0.0001, ***, *P* < 0.001, **, *P* < 0.01.

CH is one of the leading causes of HF and is regulated by the master regulator mTORC1. Since Pip4k2c negatively impacts the immune system by suppressing mTORC1,^[^ ^23^ ^]^ we hypothesized that human PIP4K2C levels would be higher in heart tissue taken from the left ventricular myocardium of a Non‐Failing (NF) heart than in samples taken from patients with CF or HF. To test our hypothesis and to investigate the role of PIP4K2C in heart diseases, we analyzed PIP4K2C mRNA or protein levels in the left ventricle (LV) of hearts taken from patients with CH, HF, or NF (**Figure** **1**). We found that heart tissue from patients with CH and HF had significantly lower PIP4K2C mRNA levels than tissue from NF hearts (Figure 1a). In parallel, LV heart tissue taken from patients with CH or HF contained notably less PIP4K2C protein, as shown by western blot, than tissue from NF hearts (Figure 1b,c).

To evaluate mouse Pip4k2c mRNA and protein expression in CH, CF, and HF, we used a transverse aortic constriction (TAC) mouse model (pressure overload). TAC was performed, and cardiac tissue was collected 4, 7, and 21 days later (Figure 1d). We observed, similar to the human results, significantly lower Pip4k2c mRNA and protein levels short term post TAC injury (4 days), as compared to sham‐operated mice (Figure 1e–g). We also observed Pip4k2c expression in both CM and non‐CMs (including cardiac fibroblasts, endothelial, and smooth muscle cells) isolated from P8 mice (Figure S1a,b: Supporting Information). To analyze the distribution of Pip4k2c expression in the two cell types, we isolated CM and non‐CM from both sham‐ and TAC‐operated mice long‐term post TAC injury (7 or 21 days). We chose longer time points as TAC leads to progressive cardiac disease. We saw continuing reduction of Pip4k2c mRNA levels in both CM and non‐CM (Figure 1h) at different time points following TAC. Taken together, our data suggest that all heart cell types express Pip4k2c, which is significantly lower in heart cells from patients with CH or HF and post‐TAC mice.

### 2.2 Loss of Pip4k2c Induced Cardiac Hypertrophy and Fibrosis Post TAC in Mice

**Figure 2:**
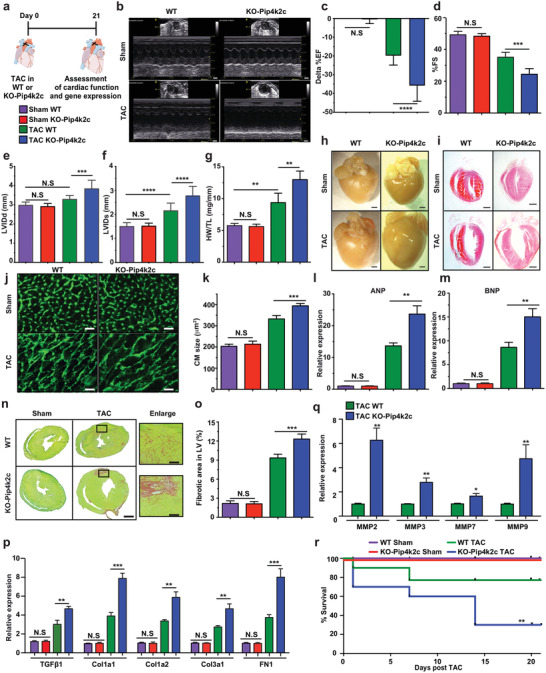
Loss of Pip4k2c enhances formation of cardiac hypertrophy and fibrosis post TAC injury. a) Experimental timeline to evaluate cardiac function and outcome in Pip4k2c^−/−^ (KO‐Pip4k2c) and Pip4k2c^+/+^ littermate controls (WT) in a TAC mouse model. b) Representative echocardiography image of left ventricle 21 days post sham or TAC injury in WT or KO‐Pip4k2c. c–f) Echo evaluation of delta % left ventricular ejection fraction (c), fractioning shorting (d), LVIDd (e), and LVIDd (f) 21 days post sham or TAC injury in WT or KO‐Pip4k2c (*n* = 10). g) Heart weight to tibia length 21 days post sham or TAC injury in WT or KO‐Pip4k2c (*n* = 10). h,i) Representative images of whole heart (h) and H&E staining (i) 21 days post sham or TAC injury in WT or KO‐Pip4k2c. j) Representative images of wheat germ agglutinin (WGA) staining to evaluate CM size (cross‐sectional area) 21 days post sham or TAC injury in WT or KO‐Pip4k2c. k) Quantitative analysis of j (*n* = 8). l,m) qPCR analysis of hypertrophic markers 21 days post sham or TAC injury in WT or KO‐Pip4k2c (*n* = 5). n) Representative images of Sirius red / fast green indicating fibrotic area 21 days post sham or TAC injury in WT or KO‐Pip4k2c. o) Quantitative analysis of n (*n* = 8). p,q) qPCR analysis of TGF*β*1 and its downstream target genes (p, *n* = 5) or different matrix metalloprotease (MMP) genes (q, *n* = 5). r) Survival curve after TAC injury in WT or KO‐Pip4k2c (*n* = 10). One‐way ANOVA, Bonferroni post‐hoc test for (C‐F, J‐L, and N&O). Unpaired two‐tailed *t*‐test for p. Mantel‐Cox log‐rank test (Q). ***, *P* < 0.001, **, *P* < 0.01, *, *P* < 0.05, N.S., Not Significant. Scale bar = 1 mm (b, h, I , n), 50 µm (j,n enlarge image).

To study the role of Pip4k2c in cardiac development and diseases, we used germline‐deleted Pip4k2c^−/−^ (KO‐Pip4k2c) mice. During mouse heart development, we saw no significant change in mouse viability, growth, weight, heart weight, heart weight to body weight ratio (HW/BW), or CM number in E18 KO‐Pip4k2c mice versus littermate control mice (WT) (Figure S2, Supporting Information). In order to analyze the effect of Pip4k2c on cardiac function, CH, and CF, we used a TAC mouse model in 8‐ to 12‐week‐old KO‐Pip4k2c or WT mice and compared them to sham‐operated KO‐Pip4k2c or WT mice 21 days after the procedures (**Figure** **2**a). We show that KO‐Pip4k2c mice have impaired cardiac function, as indicated by reduced contractility (Figure 2b), % ejection fraction (%EF, Figure 2c), % fractional shortening (%FS, Figure 2d), LV Internal Diastolic Diameter (LVIDd, Figure 2e), and Internal Systolic Diameter (LVIDs, Figure 2f) compared to WT or sham‐operated controls. Moreover, hearts and CM from KO‐Pip4k2c animals that underwent TAC were significantly larger than those from their WT counterparts and sham‐operated controls; these differences can be seen in heart weight to tibia length (HW/TL) ratios (Figure 2g), whole heart picture or H&E staining (Figure 2h,i), and wheat germ agglutinin (WGA) immunostaining and CM size evaluation (Figure 2j,k). This increased CM size positively correlated with upregulated classic markers of cardiac hypertrophy, such as atrial natriuretic peptide (ANP) and brain natriuretic peptide (BNP) (Figure 2l,m) in hearts of KO‐Pip4k2c mice after TAC in comparison to WT TAC or sham‐operated mice. Importantly, Pip4k2c deletion did not significantly change the expression of other Pip4k2s (Pip4k2a and Pip4k2b) in the heart (Figure S3, Supporting Information). To further evaluate CM size and structure, we isolated adult CM from KO‐Pip4k2c or WT hearts 21 days after TAC (Figure S4a, Supporting Information). Our analysis demonstrated that CM from KO‐Pip4k2c mice are indeed larger and have higher hypertrophy marker levels than WT CM, 21 days post TAC (Figure S4b–d: Supporting Information); CM sarcomere structures did not differ significantly (Figure S4e, Supporting Information). To evaluate CF in the differently treated groups, we used Sirius red / Fast green staining and qPCR evaluation for fibrosis markers and TGF*β*1 (Figure 2n–q). We observed significantly elevated fibrosis (Figure 2n,o), TGF*β*1, target gene expression (Figure 2p), and matrix metalloproteases (MMP) (Figure 2q) in KO‐Pip4k2c hearts following TAC as compared to TAC‐operated WT or sham‐operated controls. Due to this increased CH and CF, the KO‐Pip4k2c TAC mice had notably lower survival rates than the WT TAC mice or sham‐operated KO‐Pip4k2c or WT mice (Figure 2r). Importantly, FACS analysis of immune cell distribution in KO‐Pip4k2c or WT hearts 21 days post MI showed no significant differences in immune cell infiltration into the heart (Figure S5, Supporting Information).

### 2.3 Transiently Upregulating Pip4k2c in the Heart, Using modRNA, Reverses CH and CF in a TAC Mouse Model

**Figure 3:**
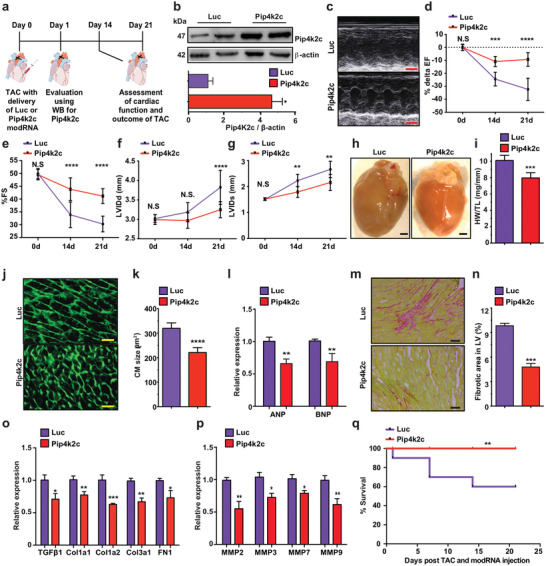
Pip4k2c modRNA elevates Pip4k2c levels, attenuating cardiac hypertrophy and fibrosis post TAC injury. a) Experimental timeline to evaluate Pip4k2c modRNA expression and cardiac function in TAC mouse model. b) Western blot of Luc (control) or Pip4k2c modRNA expression in mouse hearts 24 hours post TAC (*n* = 2) and quantitative analysis thereof (*n* = 2). c. Representative echocardiography image of left ventricle 21 days post TAC injury and delivery of Luc or Pip4k2c modRNA. d–g) Echo evaluation of delta % left ventricular ejection fraction (d), fractioning shorting (e), LVIDd (f), and LVIDs (g) before (day 0), 14, or 21 days post TAC injury and delivery of Luc or Pip4k2c modRNA (*n* = 8). h) Representative images of whole heart 21 days post TAC injury and delivery of Luc or Pip4k2c modRNA. i) Heart weight to tibia length 21 days post TAC injury and delivery of Luc or Pip4k2c modRNA (*n* = 8). j) Representative images of WGA staining to evaluate CM size (cross‐sectional area) 21 days post TAC injury and delivery of Luc or Pip4k2c modRNA. k) Quantitative analysis of j (*n* = 8). l) qPCR analysis of hypertrophic markers 21 days post TAC injury and delivery of Luc or Pip4k2c modRNA (*n* = 5). m) Representative images of Sirius red / fast green to evaluate fibrotic area 21 days post TAC injury and delivery of Luc or Pip4k2c modRNA. n) Quantitative analysis of m (*n* = 8). o,p) qPCR analysis of TGF*β*1 and its downstream target genes (o, *n* = 5) or different matrix metalloprotease (MMP) genes (p, *n* = 5). q) Survival curve of mice post TAC injury and delivery of Luc or Pip4k2c modRNA (*n* = 10). Unpaired two‐tailed *t*‐test for c, e–g, i, k,l, n–p. Mantel‐Cox log‐rank test was used in q. ***, *P* < 0.001, **, *P* < 0.01, *, *P* < 0.05. Scale bar = 1 mm (c,h), 50 µm (j), 100 µm (m).

As Pip4k2c loss has detrimental effects in the heart post TAC, we hypothesized that transiently upregulating Pip4k2c in a TAC setting would benefit CH, CF, and overall heart function. To test this, we used the novel modRNA delivery platform to raise Pip4k2c (**Figure** **3**a). As we have shown previously,^[^ ^24^, ^25^, ^26^, ^27^, ^28^, ^29^, ^30^, ^31^ ^]^ modRNA can translate any open reading frame for 8–12 days in both CM and non‐CMs without eliciting an immune response or compromising the host genome. Since modRNA has never before been used for gene delivery in a TAC model, we first used Cre recombinase (Cre) modRNA and R26mTmG mice to evaluate modRNA biodistribution in the mouse heart following TAC (Figure S6a, Supporting Information) and found that modRNA‐delivered Cre led to more than 40% LV transfection in a TAC model (Figure S6b,c: Supporting Information). We also showed, in vivo using western blot analysis (Figure 3b–e) and in vitro using immunostaining (Figure S7a,b: Supporting Information), that Pip4k2c modRNA can translate for at least 10 but not 21 days in vivo in both CM and non‐CM. Further, we demonstrated that a single dose of Pip4k2c modRNA at the time of injury beneficially affects cardiac function (Figure 3c), represented by increased %EF (Figure 3d), %FS (Figure 3e), LVIDd, and LVIDs (Figure 3f,g) 21 days post TAC, as compared to mice treated with Luc modRNA (control). Interestingly, neither diastolic nor systolic left ventricular posterior wall diameter significantly changed (Figure S8, Supporting Information), while both heart and CM size were notably smaller in Pip4k2c modRNA‐treated hearts versus control, as can be seen in whole heart images (Figure 3h), HW/TL (Figure 3i), WGA immunostaining, and CM size evaluation (Figure 3j,k) 21 days after TAC. Additionally, decreased CM size positively correlated with lower expression of CH markers (ANP and BNP) in Pip4k2c modRNA‐treated hearts than in controls 21 days post TAC (Figure 3l). We also evaluated CF following Pip4k2c modRNA delivery and found reduced fibrosis area (Figure 3m,n), inhibited expression of TGF*β*1 and its target genes (Figure 3o), and fewer matrix MMP's (Figure 3p) 21 days after TAC in Pip4k2c modRNA‐treated hearts as compared to controls. Decreased CH and CF after TAC and Pip4k2c modRNA treatment significantly raised mouse survival (Figure 3q). Taken together, our data indicate that a single dose of Pip4k2c modRNA in a mouse TAC model significantly reversed CH and CF 21 days post injury, thereby improving cardiac function and survival.

### 2.4 Pip4k2c Regulates CH in CM through mTORC1 Signaling

**Figure 4:**
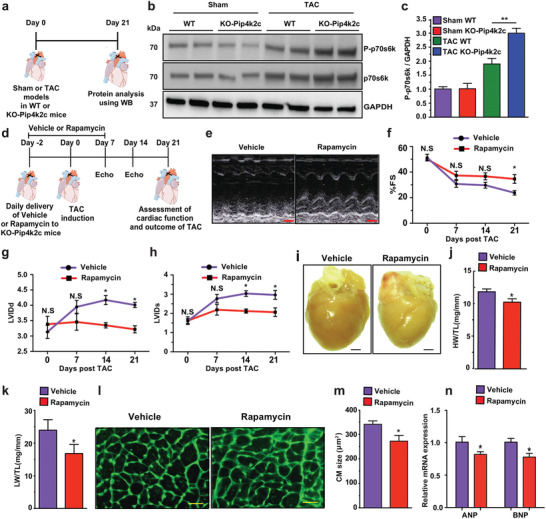
Pip4k2c attenuates cardiac hypertrophy and fibrosis via mTORC1 and TGF*β*1, respectively. a) Experimental timeline to evaluate mTORC1 pathway activation. b) Western blot analysis of phospho‐p70s6k and p70s6k protein expression in WT or KO‐Pip4k2c 21 days post sham or TAC injury. c) Quantitative analysis of b (*n* = 2). d) Experimental timeline to evaluate the effect of vehicle or rapamycin small molecule on cardiac function, CH, and CF. e) Representative echocardiography image of left ventricle 21 days post TAC injury and delivery of vehicle or rapamycin. f–h) Echo evaluation of fractioning shorting (f), LVIDd (g), and LVIDd (h) 21 days post TAC injury and delivery of vehicle or rapamycin (*n* = 4). i) Representative images of whole heart 21 days post TAC injury and delivery of vehicle or rapamycin. j,k) Heart weight (j) or lung weight (k) to tibia length 21 days post TAC injury and delivery of vehicle or rapamycin (*n* = 4). l) Representative images of WGA staining to evaluate CM size (cross‐sectional area) 21 days post TAC injury and delivery of vehicle or rapamycin. m. Quantitative analysis of l (*n* = 4). n) qPCR analysis of ANP and BNP 21 days post TAC injury and delivery of vehicle or rapamycin (*n* = 3). One‐way ANOVA, Tukey's Multiple Comparison Test were used in c. Two‐way ANOVA, Bonferroni post‐hoc test were used in f–h. An unpaired two‐tailed *t*‐test was used for j,k, m,n, p. *, *P* < 0.05, N.S., Not Significant. Scale bar = 1 mm (e,i), 50 µm (l).

To analyze the molecular pathways Pip4k2c induces to prevent CH following TAC, we isolated protein from WT or KO‐Pip4k2c mice post sham or TAC injury (**Figure** **4**a). As CH is highly regulated via the mTOR1 pathway^[^ ^17^, ^21^, ^32^, ^33^, ^34^ ^]^ and was previously shown to be regulated by Pip4k2c in immune cells,^[^ ^23^ ^]^ we used western blot to evaluate phosphorylated and non‐phosphorylated ‐p70s6k (phosphorylated ribosomal protein S6 kinase beta‐1 (S6K1)) protein, which is a known mTORC1 pathway marker. 21 days after TAC, phosphorylated p70s6k was significantly higher in KO‐Pip4k2c mice than WT and sham‐operated WT or KO‐Pip4k2c mice (Figure 4b,c). This elevated phospho‐p70s6 kinase response is known to be pro‐hypertrophic, suggesting that Pip4k2c negatively regulates the mTORC1 pathway in the heart following TAC.

Next, we used rapamycin (a well‐known mTORC1 chemical inhibitor)^[^ ^18^, ^35^ ^]^ to further study the mTORC1 molecular pathway in KO‐Pip4k2c mice using the TAC model. We administrated rapamycin or vehicle every day from 2 days before the TAC procedure till day 7 (Figure 4d). This treatment partially reduced the detrimental effect of KO‐Pip4k2c, as compared to KO‐Pip4k2c mice treated with vehicle, 21 days post TAC. More specifically, rapamycin treatment improved cardiac function, as demonstrated by significantly increased %FS 21 days after TAC (Figure 4e,f) and notably reduced LVIDd and LVIDs (Figure 4g,h) 14 and 21 days post TAC, compared to KO‐Pip4k2c mice treated with vehicle. Furthermore, rapamycin treatment leads to smaller heart or lung weight to tibia length ratios (HW/TL or LW/TL, Figure 4i–k). Using WGA immunostaining and CM measurements, we determined that KO‐Pip4k2c mice treated with rapamycin, versus vehicle, had significantly smaller CM 21 days after TAC injury (Figure 4l,m). Finally, we observed that rapamycin treatment distinctly reduced hallmark cardiac hypertrophy markers 21 days post TAC (Figure 4n). These data suggest that delivering rapamycin to the KO‐Pip4k2c heart beneficially reduces CH following TAC.

### 2.5 Pip4k2c Regulates CF in Cardiac Fibroblasts through TGF*β*1 Signaling

**Figure 5:**
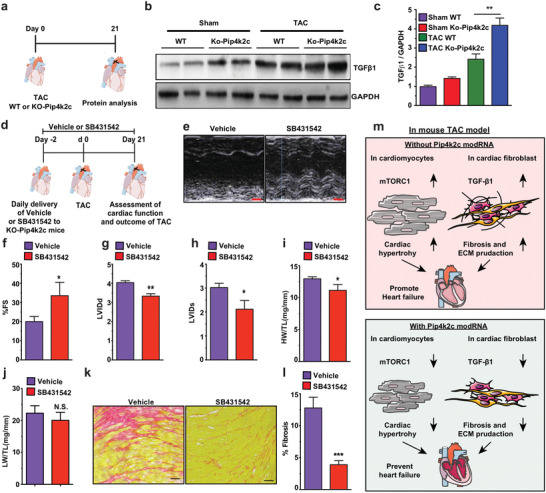
Pip4k2c attenuates cardiac fibrosis via the TGF*β*1 pathway. a) Experimental timeline to evaluate TGF*β* pathway activation. b) Western blot analysis of TGF*β* protein expression in WT or KO‐Pip4k2c 21 days post sham or TAC injury. c) Quantitative analysis of b (*n* = 2). d) Experimental timeline to evaluate the effect of vehicle or SB4311542 small molecule on cardiac function and CF. e) Representative echocardiography image of left ventricle 21 days post TAC injury and delivery of vehicle or SB4311542. f–h) Echo evaluation of fractioning shorting (f), LVIDd (g), or LVIDd (h) 21 days post TAC injury and delivery of vehicle or SB4311542 (*n* = 3). i) Heart weight to tibia length 21 days post TAC injury and delivery of vehicle or SB4311542 (*n* = 3). j) Lung weight to tibia length 21 days post TAC injury and delivery of vehicle or SB4311542 (*n* = 3). k) Representative images of Sirius red / fast green to evaluate fibrotic area 21 days post TAC injury and delivery of vehicle or SB4311542. l) Quantitative analysis of k (*n* = 3). m) A proposed model for regulating Pip4k2c modRNA in CH and CF by inhibiting mTORC1 and TGF*β*1 post TAC injury. One‐way ANOVA, Tukey's Multiple Comparison Test were used in c. An unpaired two‐tailed *t*‐test was used for f‐j, l, p. *, *P* < 0.05, N.S., Not Significant. Scale bar = 1 mm (e), 50 µm (k).

In order to investigate how Pip4k2c curtails CF post TAC, we isolated protein from WT or KO‐Pip4k2c sham‐ or TAC‐operated mice (**Figure** **5**a). As CF is highly regulated via the TGF*β*1 pathway,^[^ ^36^, ^37^, ^38^, ^39^, ^40^ ^]^ and because our loss‐ and gain‐of‐function studies (Figures 2 and 3) indicate Pip4k2c regulates TGF*β*1, we used western blot to evaluate TGF*β*1 21 days after TAC. Our findings show that TGF*β*1 was significantly higher in KO‐Pip4k2c mice than WT post sham or TAC injury (Figure 5b,c), suggesting that Pip4k2c negatively regulates the TGF*β*1 pathway in the heart following TAC.

Next, we administered TbetaR1/ALK5 inhibitor (SB431542) daily to further study the TGF*β*1 molecular pathways in KO‐Pip4k2c mice undergoing TAC (Figure 5d). We showed that SB4311542, like rapamycin, partially reduced the detrimental effect of KO‐Pip4k2c. More specifically, SB4311542 treatment improved cardiac function, as demonstrated by significantly increased %FS (Figure 5e,f) and notably diminished LVIDd and LVIDs (Figure 5g,h) 21 days after TAC, as compared to KO‐Pip4k2c mice treated with DMSO vehicle. CF evaluation following delivery of SB4311542 or vehicle revealed smaller heart but not lung weight to tibia length ratios (HW/TL or LW/TL, Figure 5i,j) accompanied by remarkably decreased fibrosis area (12.5% to 4% fibrosis in the LV, Figure 5k,l) in KO‐Pip4k2c mice treated with SB4311542, compared to DMSO vehicle, 21 days post TAC. These results suggest that SB4311542 delivery in KO‐Pip4k2c hearts after TAC can partially compensate for the loss of Pip4k2c, which induces fibrosis through the TGF*β*1 pathway. Our data suggest that Pip4k2c suppresses CH (via mTORC1 in CM) and CF (via TGF*β*1 in cardiac fibroblasts), both of which can lead to HF. Using a TAC mouse model, we demonstrated that Pip4k2c modRNA could therapeutically attenuate detrimental effects and promote better cardiac function and survival (Figure 5m).

### 2.6 N‐Terminal Motif (VMLLPDD) on Pip4k2c is Directly Responsible for its Suppression of the TGF*β*1 Pathway

**Figure 6:**
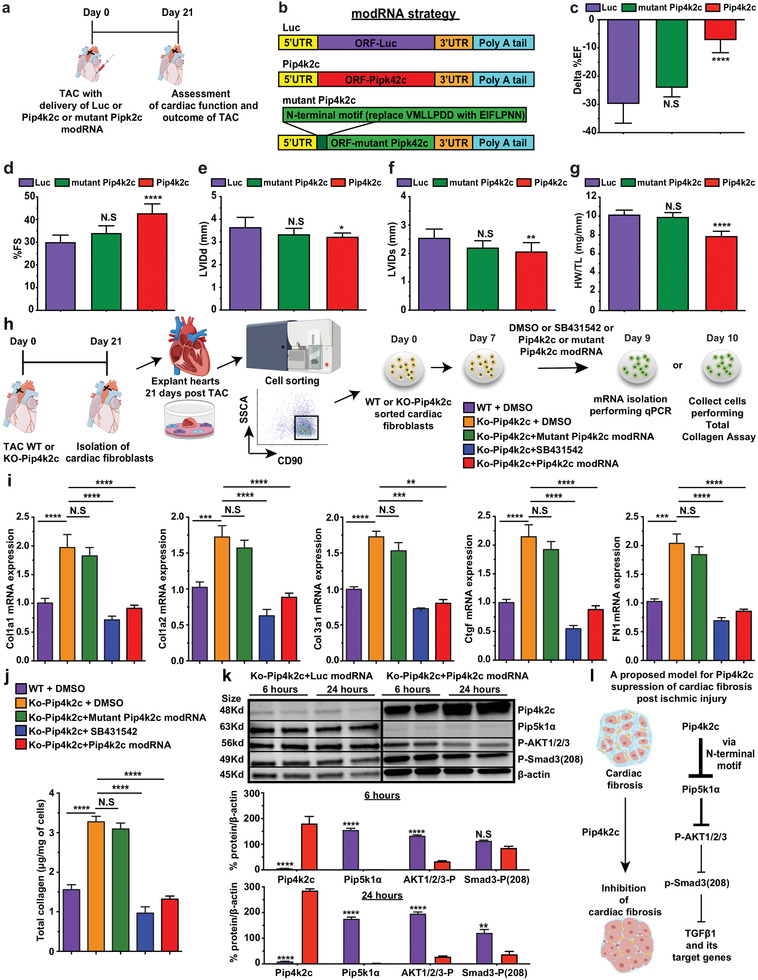
Pip4k2c suppresses the TGF*β*1 pathway via its N‐terminal motif (VMLLPDD), which inhibits Pip5k1*α*, phospho‐AKT 1/2/3, and phospho‐Smad3. a) Experimental plan to evaluate the impact of mutant Pip4k2c (original N‐terminal motif VMLLPDD replaced with EIFLPNN) on cardiac function in the TAC mouse model. b) modRNA strategy for the three modRNA tested. c–f) Echo evaluation of delta % left ventricular ejection fraction (c), fractioning shorting (d), LVIDd (e), and LVIDd (f) 21 days post TAC injury and delivery of Luc, Pip4k2c, or mutant Pip4k2c modRNA (Luc‐, *n* = 3; Pip4k2c‐ *n* = 3; mutant Pip4k2c modRNA‐ *n* = 6). g) Heart weight to tibia length 21 days post TAC injury and delivery of Luc or Pip4k2c modRNA (Luc‐, *n* = 3; Pip4k2c‐ *n* = 3; mutant Pip4k2c modRNA‐ *n* = 6). h) Experimental plan to evaluate Pip4k2c's effect on fibrosis marker expression in cardiac fibroblasts isolated and sorted (for the fibroblastic marker CD90) from WT or KO‐Pip4k2c hearts 21 days post TAC injury and treated with DMSO (control), TbetaR1/ALK5 inhibitor (SB431542), Pip4k2c, or mutant Pip4k2c modRNA i) qPCR analysis of fibrosis markers placed downstream of TGF*β*1 following different treatments (*n* = 3). j) Estimation of total collagen in cells 3 days after different treatments (*n* = 3). k) Western blot analysis for isolated KO‐Pip4k2c cardiac fibroblasts for Pip4k2c modRNA's effects on the expression of Pip5k1*α*, phospho‐AKT 1/2/3, and phospho‐Smad3. l) A proposed model for the molecular pathway regulated by Pip4k2c's N‐terminal motif on TGF‐*β*1 in cardiac fibroblasts. One‐way ANOVA, Tukey's Multiple Comparison Test were used in (c–g) and (i,j). An unpaired two‐tailed *t*‐test was used for k. ****, *P* < 0.0001, ***, *P* < 0.001, **, *P* < 0.01. *, *P* < 0.05 N.S., Not Significant.

To map the molecular pathways Pip4k2c engenders to prevent CF after TAC, we prepared mutant Pip4k2c modRNA in which the N‐terminal motif (VMLLPDD) was replaced with the mutant motif EIFLPNN. This mutation was previously shown to abolish Pip4k2c's ability to inhibit the mTOR pathway.^[^ ^16^ ^]^ We then used the TAC mouse model to compare Pip4k2c and mutant Pip4k2c modRNA (**Figure** **6**a,b). Unlike Pip4k2c, mutant Pip4k2c did not significantly change cardiac function (Figure 6c–f) or CH (Figure 6g), in comparison to Luc control modRNA. In addition, we isolated and sorted (for the fibroblastic marker CD90) cardiac fibroblasts from WT or KO‐Pip4k2c hearts 21 days post TAC and treated them with DMSO (control), SB431542, Pip4k2c modRNA, or mutant Pip4k2c modRNA. Next, we evaluated the fibroblasts’ expression of fibrosis markers, which are the direct targets of TGF*β*1 (2 days after treatment, Figure 6i), total collagen (3 days after treatment, Figure 6j), or proliferation (5 days after treatment, Figure S9: Supporting Information). These analyses showed that, similarly to SB431542, Pipk2c can inhibit TGF*β*1 target genes, collagen production, and cardiac fibroblast proliferation, while mutant Pip4k2c cannot, and behaves much like KO‐Pip4k2c cardiac fibroblasts. Recent publications have demonstrated that the Pip4k2c N‐terminus motif interacts with and suppresses Pip5k1*α*, thereby hindering phospho‐AKT in a prostate cancer cell line.^[^ ^16^, ^41^ ^]^ Furthermore, inhibiting phosphorylated phospho‐AKT decreases Smad3 phosphorylation on site Ser208 (Smad3‐Ser208); such phosphorylation is crucial for TGF*β*1 expression and pro‐fibrotic activity.^[^ ^42^ ^]^ We therefore wanted to evaluate if phospho‐AKT and phospho‐smad3 (208) play a role in inhibiting Pip4k2c on TGF*β*1 in the heart. To accomplish this, we collected KO‐Pip4k2c cardiac fibroblasts 6 or 24 hours post transfection with Luc or Pip4k2c modRNA, isolated and sorted the fibroblasts, and then evaluated them using western blot. Our data show significantly elevated Pip4k2c that leads to remarkably reduced Pip5k1*α*, phospho‐AKT 1/2/3, and phospho‐Smad3 (208) after transfection with Pip4k2c modRNA as compared to control. These findings indicate that Pip4k2c negatively regulates TGF*β*1 via its N‐terminal motif (VMLLPDD); reduces Pip5k1*α*, phospho‐AKT 1/2/3, and phospho‐Smad3 6 in cardiac fibroblasts; and thereby lowers their proliferation and fibrotic activity (Figure 6l).

Overall, our data demonstrate that Pip4k2c modRNA inhibits the mTORC1 pathway in CMs and the TGF*β*1 pathway in cardiac fibroblasts post TAC, thereby reducing CH and CF, resulting in better cardiac function and survival following TAC injury (Figure S10, Supporting Information).

## 3 Discussion

There is a great need to identify new potential treatments for CH and CF in order to prevent HF. Understanding the mechanisms of action that induce CH and CF, and how to control those mechanisms, may help uncover previously unknown therapeutic targets that can be used to treat patients with CH and HF. To date, the role of Pip4k2c in heart development and disease has not been studied. Though Pip4k2c may not have a regulatory role during cardiac development (Figure 2 and Figure S2: Supporting Information), human PIP4K2C mRNA and protein levels are significantly reduced in the LV of patients with CF and HF (Figure 1). Using loss‐ and gain‐of‐function studies, we demonstrate that mouse Pip4k2c affects CH and CF, thereby influencing cardiac function and survival in a TAC mouse model (Figures 2 and 3). From a mechanistic point of view, these results confirm that Pip4k2c, via its N‐terminal motif (VMLLPDD), inhibits mTORC1 in CMs. Further, our work is the first to show that Pip4k2c, via its N‐terminal motif, inhibits TGF*β*1 in cardiac fibroblasts by suppressing Pip5k1*α*, phospho‐AKT 1/2/3, and phospho‐Smad3; prevents CH (of CM) and CF (in cardiac fibroblasts); and improves TAC injury outcomes (Figures 4, 5, 6 and Figure S9: Supporting Information).

PIP4Ks (including PIP4k2a, PIP4k2b, and PIP4k2c) play important roles in insulin production^[^ ^16^ ^]^ and immune response.^[^ ^23^ ^]^ The loss of PIP4k2a or PIP4k2b limits the mTORC1 pathway, which Pip4k2c induces.^[^ ^16^ ^]^ Here we show that PIP4k2c, via its N‐terminal motif, limits TGF*β*1, a master regulator gene with respect to CF,^[^ ^40^ ^]^ and its downstream target genes (Figures 2, 3, 4, 5, 6). TGF*β*1, which is pro‐fibrotic, increases after cardiac ischemic injury and can lead to CM cell death.^[^ ^39^, ^40^ ^]^ TGF*β*1 signaling involves activation via ligand‐receptor recognition with TGF*β* receptor type II and TGF*β* receptor type I (TGF*β*RI AKA as ALK5) recruitment. In the canonical TGF*β*1 signaling pathways, Smads 2 and 3 are activated via ALK5 while Smads 1 and 5 are activated via Smads 1 and 5. The two Smads pairs create a complex that binds to Smad 4, which leads the complex into the nucleus to promote expression of the TGF*β*1, extracellular matrix, matrix metalloproteases, and myofibroblast‐related genes.^[^ ^39^, ^40^, ^43^, ^44^, ^45^ ^]^ Several strategies to inhibit TGF*β*1 signaling have been tested, with limited success.^[^ ^36^, ^37^, ^46^, ^47^ ^]^ Therefore, identifying new targets that can control TGF*β*1 signaling may have important therapeutic potential in heart disease. It is not clear if PIP4k2a and PIP4k2b suppress mTORC1 or TGF*β*1 in the mouse TAC model. The fact that it can simultaneously suppress two fundamental pathways, mTORC1 in CM and TGF*β*1 in cardiac fibroblasts, posits PIP4k2c as a key target for regulating CH and CF in HF patients. As the TGF*β*1 and, in some cases, mTORC1 pathways are crucial to other fibrotic diseases, such as pulmonary fibrosis and chronic renal fibrosis, it will be vital to evaluate the effect of PIP4k2c in these settings. Further, we show that immune cell composition in KO‐Pip4k2c mice is similar to that in WT mice 21 days post TAC (Figure S5, Supporting Information). Together with our Pip4k2c modRNA data, this strongly suggests that Pip4k2c impacts cardiac cells directly and not via suppressing the immune system.

Our current project also shows that the small‐molecule drugs rapamycin and SB4311542 partially overcome Pip4k2c loss in the heart following TAC injury (Figures 4 and 5). As rapamycin and SB4311542 are well‐known inhibitors of mTORC1 and TGF*β*1, respectively, and have been widely used in the clinic, it would be intriguing to determine whether or not they can be replaced with Pip4k2c modRNA. The longer pharmacokinetics of Pip4k2c modRNA allow it to be administered in a single dose, without known toxicity, and its activity localizes only in the target tissue (i.e., the heart), whereas small‐molecule drugs need daily administration, have some toxicity (e.g., rapamycin has known toxicity in the pancreas^[^ ^48^ ^]^), and are active in all tissues and organs within a treated individual.

Our study transfected modRNA in vitro using RNAiMAX, while in vivo we used naked modRNA transfected with sucrose‐citrate buffer. In vitro, naked modRNA transfection was not successful, as the modRNA floated over the cells (data not shown). In vivo, as we have shown previously,^[^ ^24^ ^]^ naked modRNA translates 20 times higher than capsulated modRNA using commercially available nanoparticles. Furthermore, in moderna and AstraZeneca Phase 2a human clinical trials in Finland (AZD8601), patients with ischemic heart disease receive VEGFA modRNA delivered naked in citrate‐sucrose buffer.^[^ ^49^ ^]^ The mechanism of action that allows naked modRNA to enter the cell, as both modRNA and the cell membrane are negatively charged, is not yet clear. Additionally, modRNA was recently shown to be a safe, effective Spike protein gene delivery system for vaccination against SARS‐CoV‐2 to prevent COVID‐19^[^ ^50^, ^51^ ^]^ in humans. Both companies (e.g., moderna and Pfizer‐BioNTech) producing these vaccines chose to capsulate Spike modRNA in nanoparticles to insure consistent delivery. While capsulated modRNA can lead to lower translation, it also protects against RNase cleavage in the blood. In our published cardiac studies^[^ ^24^, ^26^, ^27^, ^28^, ^52^, ^53^, ^54^, ^55^ ^]^ and our current work, we use a dissecting microscope to perform intramyocardial injections, allowing us to avoid injecting the naked modRNA into blood vessels, which can lead to it's cleavage.

Overall, modRNA is a transient, safe, controlled, dose‐dependent gene delivery method that has thus far been used to upregulate a gene of interest (VEGF‐A,^[^ ^27^, ^56^ ^]^ IGF1,^[^ ^28^ ^]^ mutant hFSTL1,^[^ ^26^ ^]^ aYAP,^[^ ^57^ ^]^ AC,^[^ ^29^ ^]^ or Pkm2^[^ ^30^ ^]^) in I/R or MI models in order to promote cardiovascular effects, induce epicardial fat, or support CM proliferation. Here, in a TAC model, we show that modRNA has high biodistribution, as over 40% of the LV is transfected (Figure S6, Supporting Information), and that Pip4k2c modRNA pharmacokinetics show expression for more than 10 days (Figure S7, Supporting Information). Indeed, a single dose of Pip4k2c modRNA engenders cardioprotection and reduces CH and CF in a TAC mouse model, with translational capacity. It is not yet clear if a single dose is sufficient for long‐lasting effects in mice or larger animals. As intramyocardially injected Pip4k2c modRNA upregulates Pip4k2c protein for 10–12 days (Figure S7C–E, Supporting Information), we should seek new, minimally invasive routes to deliver modRNA into the injured heart.

Because the modRNA gene delivery platform is currently being evaluated for cardiac use in a clinical setting, we aim to apply Pip4k2c modRNA in a large animal model to pave the way toward human clinical studies.

## 4 Experimental Section

##### Mice

All animal procedures were performed under protocols approved by the Icahn School of Medicine at Mount Sinai Institutional Animal Care and Use Committee (IACUC). Male and female C57BL/6J or CFW mice were used. Different modRNAs (100 µg per heart) were injected directly into the myocardium during open chest surgery. 3 to 10 animals were used for each experiment. The Pip4k2c^−/−^ mice were purchased from Jackson Laboratories (Pip4k2c^tm1b(KOMP)Wtsi^). For long‐term survival, 8‐ to 10‐week‐old C57BL/6J or Pip4k2c^−/−^ mice were treated without or with Luc or Pip4k2c modRNAs (*n* = 10) post TAC and allowed to recover for the specified time in the animal facility. Deaths were monitored and documented. Tissues were harvested from C57BL/6J or Pip4k2c^−/−^ mice on E18 for analysis. Mouse husbandry was carried out according to the protocol approved by the IACUC. Oligonucleotide sequences for genotyping the above mouse lines: Pip4k2c ‐F‐ CACACCTCCCCCTGAACCTGAAAC, Pip4k2c ‐R‐ AGCCGCTGGGGCCAGATGAT.

##### Human Left Ventricle (LV) Samples

Human heart tissue specimens were obtained from the National Disease Research Interchange through the Human Tissues and Organs for Research Resource program. HF heart samples were obtained from patients with end‐stage heart failure undergoing heart transplantation. Normal or CH heart samples were procured from donors who died in accidents and who had either CH or hearts unsuitable for transplantation for non‐cardiac reasons (i.e., healthy). Normal and CH conditions were defined by cardiac pathology. The control samples were obtained from donors with uninjured normal hearts: 1) Caucasian, Male, 54 years old, died on 4/19/1994; 2) Caucasian, Male, 58 years old, died on 9/24/2000; 3) Caucasian, Female, 68 years old, died on 9/1/1993; 4) Caucasian, Male, 53 years old, died on 9/24/1997; 5) Hispanic, Female, 39 years old, died on 11/26/1998. Samples of CH hearts, defined by cardiac pathology using known criteria,^[^ ^58^, ^59^ ^]^ were obtained from: 1) Afro‐American, Male, 47 years old, died on 5/22/2000; 2) Caucasian, Female, 51 years old, died on 7/13/1995; 3) Caucasian, Male, 68 years old, died on 9/4/2000; 4) Afro‐American, Male, 37 years old, died on 11/27/2005; 5) Caucasian, Female, 58 years old, died on 7/30/2006. Samples of HF hearts were obtained from patients with either ischemic heart disease: 1) Caucasian, Female, 66 years old, died on 10/11/2000; 2) Caucasian, Male, 69 years old, died on 4/1/1993; 3) Caucasian, Male, 52 years old, died on 12/29/1993; 4) Afro‐American, Female, 62 years old, died on 10/12/2001; or dilated cardiomyopathy: 5) Caucasian, Male, 53 years old, died on 9/23/1997.

##### ModRNA Synthesis

ModRNAs were transcribed in vitro from plasmid templates (see Table S1 (Supporting Information) for a complete list of open reading frame sequences used to make the modRNA for this study) using a customized ribonucleotide blend of anti‐reverse cap analog; 3 ´‐O‐Me‐m7G (5')ppp(5')G (6 × 10^−3^ m, TriLink Biotechnologies); guanosine triphosphate (1.5 × 10^−3^ m, Life Technology); adenosine triphosphate (7.5 × 10^−3^ m, Life Technology); cytidine triphosphate (7.5 × 10^−3^ m, Life Technology); and N1‐Methylpseudouridine‐5'‐Triphosphate (7.5 × 10^−3^ m, TriLink Biotechnologies) as described previously in the recent protocol paper.^[^ ^52^ ^]^ The mRNA was purified using the Megaclear kit (Life Technology) and treated with antarctic phosphatase (New England Biolabs), followed by re‐purification using the Megaclear kit. The mRNA was quantitated by Nanodrop (Thermo Scientific), precipitated with ethanol and ammonium acetate, and resuspended in 10 × 10^−3^ m TrisHCl, 1 × 10^−3^ m EDTA.

##### ModRNA Transfection

In vivo modRNA transfection was performed as described in the recent method paper,^[^ ^24^ ^]^ using sucrose citrate buffer containing 20 µL of sucrose in nuclease‐free water (0.3 g mL^−1^), with 20 µL of citrate (0.1 m pH = 7; Sigma) mixed with 20 µL of different modRNA concentrations in saline, to a total volume of 60 µL. The transfection mixture was directly injected (three individual injections, 20 µL each) into the myocardium. For in vitro transfection, RNAiMAX transfection reagent was used (Life Technologies) according to the manufacturer's instructions.

For additional methods please see the Supporting Information.

## Conflict of Interest

The authors declare no conflict of interest.

## Supporting information

Supporting Information

## Data Availability

All modified mRNA (modRNA) vectors containing any genes of interest described in this paper will be made available to other investigators. My institution and I will adhere to the NIH Grants Policy on Sharing of Unique Research Resources. Specifically, material transfers will be made with no more restrictive terms than in the Simple Letter Agreement or the UBMTA and without reach‐through requirements. Should any intellectual property arise which requires a patent, we would ensure that the technology remains widely available to the research community in accordance with the NIH Principles and Guidelines.
