# Supplementary material for: Therapeutic Delivery of Pip4k2c‐Modified mRNA Attenuates Cardiac Hypertrophy and Fibrosis in the Failing Heart
Source: Adv Sci (Weinh). 2021 Mar 12;8(10):2004661. doi: 10.1002/advs.202004661 (PMC8132051; doi:10.1002/advs.202004661)
Supplement: Supplementary file 1 — Supporting Information [file ADVS-8-2004661-s001.pdf]

© 2021 Wiley-VCH GmbH

ADVANCED SCIENCE
Open Access

Supporting Information

for *Adv. Sci.*, DOI: 10.1002/advs.202004661

Therapeutic delivery of Pip4k2c-modified mRNA attenuates cardiac hypertrophy and fibrosis in the failing heart

*Ajit Magadum PhD, Neha Singh PhD, Ann Anu Kurian MSc , Mohammad Tofael Kabir Sharkar PhD,Nishat Sultana PhD, Elena Chepurko MDM, Keerat Kaur PhD, Magdalena M. Żak, Yoav Hadas PhD , Djamel Lebeche PhD, Susmita Sahoo PhD, Roger Hajjar MD, and Lior Zangi PhD**

Supplemental Information

Table of Contents

1) Supplemental Methods and Materials

2) Supplemental Tables

3) Supplemental Figures

4) Author Contributions

1. Supplemental Methods and Materials

Transverse Aortic Constriction (TAC) model

All surgical and experimental procedures with mice were performed in accordance with protocols approved by IACUC and the MSSM Center for Comparative Medicine and Surgery (CCMS). Thoracic aortic constriction was performed on 8-week-old male C57BL/6J or CFW mice (Charles River Laboratories), as described previously with small adaptations[4]. Mice received buprenorphine (0.1 mg/kg s.c.) 60 min before intubation and anesthesia with isoflurane. Thoracotomy was performed between the second and third rib, and the aortic arch was narrowed by a ligature over a 27 G cannula to generate trans-stenotic pressure of approximately 40 mmHg in all animal groups. In addition to undergoing the same constriction, all mice were of the same age and body weight. Mice remained in a warmed cage for 2-4 h, until completely recovered from anesthesia, under direct supervision. In sham surgery, the chest was opened, but the aorta was not ligated. Cardiac dimensions and function were analyzed

by pulse-wave Doppler echocardiography before TAC/sham surgery and before the animals were euthanized. Mice were sacrificed to determine cardiac hypertrophy, fibrosis, and other parameters. In all experiments, the surgeon was blinded to the treatment group. To assess heart histology, hearts were collected at the end of each experiment. The hearts were excised, briefly washed in PBS, perfused with perfusion buffer, weighed, and fixed in 4% PFA at 4°C overnight. The next day, hearts were washed with PBS and incubated overnight in 30% sucrose. Next, hearts were put in OCT, frozen, and stored at -80°C. The heart blocks were sectioned transversely or longitudinally at 8-9μm using a cryostat. The slides were further processed for immunostaining (see below) or histological staining with hematoxylin and eosin (H&E) and Sirius red / fast green, all performed according to standard procedures. The heart weight to tibia length ratio was measured using a standard scale at each experiment endpoint. This ratio was calculated as the heart tissue weight relative to the mouse tibia length, with weight in grams (mg) and length in mm.

Adult cardiomyocyte and non-cardiomyocyte isolation

Cardiomyocytes and non-cardiomyocytes were isolated (for Fig 1j) from adult mice hearts using Langendorff's method as previously described[60]. Briefly, hearts were excised and the aortas were cannulated and perfused with buffer A (in mM: 113 NaCl, 4.7 KCl, 0.6 KH_2PO_4, 0.6 Na_2HPO4, 1.2 $MgSO_4$, 12 $NaHCO_3$, 10 $KHCO_3$, 10 HEPES, 30 taurine). To dissociate cells, collagenase type II (Worthington) was included in the buffer. After dissociation, the cell suspension was incubated for 10 min at 37 °C, allowing the CMs to sediment. The non-cardiomyocyte-enriched supernatant

and pellet were then resuspended in buffer B (47.5 ml perfusion buffer A, 2.5 ml FCS, 62.5 ml, 10 μM $CaCl_2$) and centrifuged for 5 min (400 x g). Both CM and non-CM fractions were used to isolate mRNA.

Isolating cardiac fibroblasts

Hearts were removed from TAC mice using curved forceps, minced into small pieces (<1 mm^3 in size) using scissors, and washed with cold PBS to remove any excess blood. 2ml of fibroblast explant medium (Iscove's Modified Dulbecco's Medium + 20% FBS) was added to each heart to dissociate the tissue pieces by pipetting. Next, the tissue pieces were plated evenly on 0.1% (wt/vol) gelatin-coated 6-well plates with 1/4 adult heart per well in 0.2ml of fibroblast explant medium. The tissue was allowed to attach for 2 hours, after which 2ml of additional fibroblast explant medium was added to each well. Cells were allowed to attach for 7-10 days before undergoing fibroblast sorting.

Sorting cardiac fibroblasts

Cardiac cells were isolated by explantation and cultured for up to 7-10 days till they were ready for staining and FACS. Cells were washed with PBS and dissociated with 0.05% (wt/vol) trypsin for 5 mins. An equal volume of fibroblast explant medium was added to neutralize the trypsin. Thereafter, the cells were passed through a 40-μm cell strainer to remove any tissue fragments and then concentrated by centrifugation at 600g for 5 min at RT. Next, the cells were stained with 200μl Thy-1/APC antibody (eBioscience, cat. no. 17-0900-82) by diluting the antibody in 10% (vol/vol) FBS in PBS

at room temperature for 30-60 mins. Cells were then washed with PBS, centrifuged at 600g for 5 min at RT, kept on ice, and sorted for Thy1+ cells. Sorted Thy1+ cells were plated at a density of 10^4 cells per cm^2.

Total collagen assay

Isolated and sorted cardiac fibroblasts from WT or KO-pip4k2c mice 21 days post TAC operation were treated with DMSO, SB431542, Luc modRNA, Pip4k2c, or mutPip4k2c modRNA. 3 days later, cells were washed with PBS, then harvested, and lysates were evaluated for collagen production using a total collagen assay kit from Abcam (ab222942). The colorimetric signals were analyzed at OD 560 nm on a microplate reader according to standard procedures.

Echocardiography (ECHO)

We evaluated left ventricle dimensions and function by performing transthoracic two-dimensional echocardiography. In a double-blind study (neither the surgeon nor the echography technician was aware of the treatment), various modRNAs were injected into C57BL/6J or CFW mice (8 to 12 weeks old). Animals underwent echography on-site with a GE Cares machine (V7R5049) equipped with a 40 MHz mouse ultrasound probe. Mice were anesthetized with a mixture of 1-2% isoflurane in air, and imaging was performed on days 2 and 28 post LAD ligation. The ejection fraction and fractional shortening were calculated as percentages from the diastolic volume (EDV) and end-systolic volume (ESV) dimensions on an M-mode ultrasound scan. The following formulas were used: % ejection fraction = (EDV-ESV)/EDV*100 and % fractional

shortening = (left ventricular internal dimension at end-diastole (LVIDd) – left ventricular internal dimension at end-systolic (LVIDs))/ LVIDd*100. Echocardiograms were performed on 4-10 hearts/treatment group.

Hematoxylin and eosin (H&E) staining

H&E staining was performed according to standard procedures. The OCT frozen longitudinal heart sections were dried for 30 min to 1 hr at room temperature, then hydrated in PBS for 10 mins. The slides were kept in Hematoxylin stain for 2 mins and washed with tap water for 5 mins. Next, the sections were stained using an eosin solution for 1 min and washed with tap water for 5 mins. The slides were transferred to PBS for 5 mins. Sections were then dehydrated in 100% ethanol and xylol for 1 min each. Finally, sections were mounted. The images were taken on a bright-field microscope.

Mouse survival post TAC

For our long-term survival study, the C57BL/6J or Pip4k2c$^{-/-}$ mice (8-10 weeks old) were subjected to a survival study after either TAC operation or modRNA injection. The mice were checked every week for death or survival (Fig 2r and Fig 3q).

E18 mice study

To analyze heart development in WT or Pip4k2c$^{-/-}$ mice, we harvested the embryonic mice at E18. We determined embryonic mouse weight and heart weight and also imaged these mice and their hearts on a bright-field microscope (Fig S2).

Flow cytometry

Left ventricles of wild type and Pip4k2c KO mice were dissected at 21 days post TAC and processed according to a previously described protocol[61] with a few modifications. After isolation, cardiac cells were stained with PerCP-Cy5.5-conjugated hematopoietic lineage markers: CD19, NK-1.1, and TER-119 (1:300) together with CD45-Alexa Fluor 700, CD11b-APC, F4/80-PE-Cy7, Ly6C-BV510, Ly6G-FITC, CD3-BV570, CD4-PE-Cy5, and CD8a-BV650 (1:300). DAPI was used as a live/dead cell marker. Myeloid cells were defined as $CD45^+$ $CD11b^+$, macrophages as $CD45^+$ $CD11b^+$ $F4/80^+$ and $Ly6C^{low/interm}$, neutrophils as $CD45^+$ $CD11b^+$ $Ly6G^{hi}$, and T-cells as $CD45^+$ $CD3^+$. Data were acquired using Aurora (Cytek) Spectral Flow Cytometer. Digestion and staining protocols were as previously described[61] with a few modifications. At 14 days post TAC, hearts of wild type and Pip4k2c KO mice were perfused using 10ml of ice-cold PBS. Left ventricles were dissected and minced in 1ml of digestion mix containing 3.7μl of DNase (Sigma, D5319); 5μl of Hyaluronidase (12000 U/ml, Sigma, H3506); 10μl of Collagenase XI (12500 U/ml, Sigma, C7657); 20μl of HEPES buffer (1M, Corning, 25-060-Cl); and 45μl of Collagenase I (10000 U/ml, Sigma, C0130) in DPBS and incubated for 1 h at 37°C with agitation (750 rpm). Then, a cell suspension was triturated and filtered through a 40μm cell strainer using FACS buffer (0.5% BSA in PBS) and centrifuged at 340 x g for 7 min at 4°C. Prior to staining, Purified Rat Anti-Mouse CD16/CD32 (Mouse BD Fc Block) was added into cell suspension (1μl per $1*10^6$ cells) and incubated for 5 min at room temperature followed by an antibody mix

(CD19, NK-1.1, TER-119, CD45-Alexa Fluor 700, CD11b-APC, F4/80-PE-Cy7, Ly6C-BV510, Ly6G-FITC, CD3-BV570, CD4-PE-Cy5, and CD8a-BV650, Biolegend, at 1:300), incubated on ice for 30min, and then washed. DAPI was added to the samples as a viability cell marker 15 min before acquisition. Data were acquired using AURORA (Cytek) Spectral Flow Cytometer and analyzed using FlowJo software. A detailed list of antibodies is available in **Table S2**, and the gating strategy is shown in Figure S5.

Immunostaining heart sections

Frozen heart sections were rehydrated in PBS for 5 min, followed by permeabilization in PBS with 0.1% triton X100 (PBST) for 7 min. Slides were then treated with 3% H_2O_2 for 5 min. After three 5-minute washes with PBST, the samples were blocked with PBS + 5% Donkey normal serum + 0.1% Triton X100 (PBSST) for 2 hrs at room temperature, and primary antibodies (see a complete list of primary antibodies used for this study in **Table S3**) diluted in PBSST were added. After being incubated overnight at 4°C, slides were washed five times with PBST (4 min per wash), then incubated with a secondary antibody (Invitrogen, 1:200) diluted in PBST for 2 hours at room temperature. The samples were washed three times in PBST (5 min per wash) and stained with DAPI or Hoechst 33342 (1µg/ml) diluted in PBST for 7 min. After five 4-min washes with PBST and one 4-min wash with tap water, slides were mounted with mounting medium (VECTASHIELD) for imaging. Stained slides were stored at 4°C. All staining was performed on 3-8 hearts/group, with 2-3 sections/heart. To immunostain neonatal rat or mouse CMs following modRNA treatment, modRNA-transfected neonatal CMs were fixed on coverslips with 3.7% PFA for 15 min at room temperature,

then washed three times with PBS. Following permeabilization with 0.5% Triton X in PBS for 10 min at room temperature, cells were blocked with 5% normal goat / donkey serum + 0.5% Tween 20 for 30 min. Coverslips were incubated with primary antibodies for 1 hr in a humid chamber at room temperature, followed by incubation with corresponding secondary antibodies conjugated to Alexa Fluor 488, Alexa Fluor 647, and Alexa Fluor 555, as well as Hoechst 33342 staining for nuclei visualization (all from Invitrogen). The fluorescent images were taken on a Zeiss fluorescent microscope at 10X, 20X, and 40X magnification.

Western blot analysis

We isolated total protein from specific cells or tissues at given time points as mentioned above. In brief, equal amounts of protein were resolved using an SDS-PAG Electrophoresis system in 4%-15% Mini-PROTEAN TGX stain-free gels (Bio-Rad) and blotted onto polyvinylidene fluoride (PVDF) membranes. The membranes were blocked (5% non-fat dry milk [DM] in Tris-buffered saline (TBS; 50 mM Tris-HCl [pH 7.4], 150 mM NaCl)) for 1 hr at room temperature and then incubated with primary antibodies diluted in 5% milk in TBS overnight at 4°C. We used anti-Pip4k2c (1:1000, Proteintech, # 17077-1-AP or 1:1000, MyBioSource,MBS9203628); anti-Phospho-p70 S6 Kinase (Thr389) (P-p70s6k) (1:2,000, Cell Signaling, #9205); p70 S6 Kinase (p70s6k) (1:2000, Cell Signaling, #9202); ant- Pip5k1α (1:1000, Novus, NBP2-19833); anti-phospho-AKT 1/2/3 (1:1000, Abcam, ab192623); anti- phospho-Smad3 (208) (1:1000, Abcam, ab138659); anti-GAPDH (horseradish peroxidase [HRP] conjugate 1:3,000, Cell Signaling, #8884); and mouse monoclonal anti-β-actin (horseradish peroxidase [HRP]

conjugate 1:3000, #12262; Cell Signaling) antibodies. Anti-rabbit and anti-mouse HRP-conjugated secondary antibodies were purchased from Sigma-Aldrich. Antigen or antibody complexes were visualized with ChemiDoc Touch imaging system (Bio-Rad).

RNA isolation and gene expression profiling using real-time PCR

Total RNA was isolated using the RNeasy mini kit (Qiagen) and reverse transcribed using Superscript III reverse transcriptase (Invitrogen) according to the manufacturer's instructions. Real-time qPCR analyses were performed on a Mastercycler realplex 4 Sequence Detector (Eppendorf) using SYBR Green (Quantitect™SYBR Green PCR Kit, Qiagen). Data were normalized to *18s* expression where appropriate (endogenous controls). Fold changes in gene expression were determined by the ∂∂CT method and presented relative to internal control. PCR primer sequences are shown in **Table S4.**

Fetal, neonatal, or adult mouse CM isolation

CMs from the hearts of E18 fetal mice (Pip4k2c$^{-/-}$ or wild type) or 3- to 4-day-old neonatal Sprague Dawley rats (Jackson) were isolated as previously described[62,63]. We used multiple rounds of digestion with 0.14-mg/mL collagenase II (Invitrogen); after each, the supernatant was collected in horse serum (Invitrogen). The total cell suspension was centrifuged at 300 g for 5 min. Supernatants were discarded and cells were resuspended in DMEM (GIBCO) medium with 0.1mM ascorbic acid (Sigma), 0.5% Insulin-Transferrin-Selenium (100X), penicillin (100U/mL), and streptomycin (100μg/mL). Cells were plated in plastic culture dishes for 90 min until most of the non-CMs attached to the dish, and the CMs remained in suspension. The CMs were then

seeded at 1 × 10^5 cells/well in a 24-well plate. Isolated CMs were incubated for 48 hrs in DMEM medium containing 5% horse serum plus Ara c. After incubation, cells were transfected with varying doses of different modRNAs, as described in the text. Adult CMs were isolated from mice using standard Langendorff's method as previously described[63]. To count CMs, we averaged three different samples and three hearts/group using a hemocytometer. We counted approximately 150-200 CMs/aliquot (10ul aliquot samples taken using a wide-bore pipette from the total volume of CMs obtained following digestion). The cultured CMs were stained with α-actinin (CM, Red) antibody (abcam) and Hoechst 33342 for nuclei counts. Approximately 1x10^3 CMs were used per sample for nuclei counts, using 3-4 independent samples per group. Nuclei count was plotted as the percentage of counted CMs.

Assessing cardiomyocyte hypertrophy (CH)

To analyze CM hypertrophy, we used wheat germ agglutinin (WGA) staining. The cryo-frozen heart sections were dried for 30-60 min at room temperature and dehydrated with PBS for 10 mins. Next, we applied the WGA (50µm) for 1 hr. The sections were washed three times with PBS for 5 mins each, and the mounting medium was applied. To quantify CM size, images at 20X or 40X magnification were captured, and the ImageJ program was used to determine the area of each cell. Quantitative analyses involved counting multiple fields from three to six independent hearts/group and three sections/heart (~50 cells per field were assessed for a total ~250 cells per sample).

Sirius red / fast green staining

The Sirius red / fast green staining was performed according to standard procedures. The cryo-frozen heart sections were dried for 1 hr at room temperature and incubated with Bouin's solution at 58°C for 1 hr, then washed with tap water for 5 mins to remove the yellow color from sections. The sections were stained with 0.1% fast green for 20 mins at room temperature and rinsed in 1% acetic acid for 1 min at room temperature. Next, slides were washed with tap water for 5 mins. The sections were further stained with 0.1% Sirius red for 30 mins at RT. Slides were dehydrated sequentially by 70% ethanol for 30 sec, 100% ethanol for 1 min, and 100% xylene for 3 min, then covered with coverslips using Permount mounting medium (Fisher Chemical). The images were taken on a bright field microscope.

SB4311542 or rapamycin treatment in mice

For SB4311542 treatment, the stock solution of SB4311542 (10mM) was diluted into a vehicle in ethanol. Mice were given 10mg/kg/day of SB4311542 through i.v. injections every day for 23 consecutive days (from 2 days before TAC until the experiment ended (day 21)). For rapamycin treatment, the stock solution of rapamycin (50mg/mL) was diluted into a vehicle [5% (vol/vol) Tween-80, 5% (vol/vol) PEG 400 (polyethylene glycol, molecular weight 400)] in 1× PBS. Mice were given 3mg/kg/day of rapamycin through i.v. injections for 9 consecutive days (from 2 days before until 7 days post TAC).

Statistical analysis

Statistical significance was determined by unpaired two-tailed t-test, One-way ANOVA, Tukey's Multiple Comparison Test, Two-way ANOVA, Bonferroni post hoc test, or Log-rank (Mantel-Cox) test for survival curves, as detailed in respective figure legends. p-Value<0.05 was considered significant. All graphs represent average values, and values were reported as mean ± standard error of the mean. An unpaired two-tailed t-test was based on assumed normal distributions. To quantify different parameters, we used WGA, pH3 CMs, and Sirius red / fast green staining with results acquired from at least three heart sections/heart in mouse numbers as noted in respective figure legends.

2. Supplemental Tables

Table S1: Open reading frame sequences used for modRNA production

Gene	Open Reading Frame
Pip4k2c	atggcgtcctcctccgtccctcccgccaccgcacccgcggcagctggaggccccggcccgggattcggctt<br>cgcctccaaaaccaagaagaagcatttcgtacagcagaaagtgaaggtgttccgggccgcggacccgct<br>ggtgggcgtgttcctgtggggcgtcgctcactcgatcaatgagctcagccaggtaccgcccccagtgatgtt<br>gctgccagatgactttaaagccagctccaagatcaaggtcaacaatcactttttccatagagaaaatcttccc<br>agtcatttcaagttcaaggagtattgtccccaggtcttcaggaacctgagagatcggtttgccatcgatgatca<br>cgattacttggtgtcccttactcgaagcccccccaagcgaaactgaaggcagtgatggccgtttccttatctcct<br>atgaccgcactctggtcatcaaggaagtgtcgagcgaagatattgcggatatgcacagcaacctctccaac<br>taccaccagtacatagtgaaatgtcatggcaacacgcttctgccccagttcctgggcatgtaccgagtcagt<br>gtagaaaatgaagacagctacatgctcgtgatgcgcaatatgtttagtcatcgtcttcctgtgcataggaagta<br>tgacctcaagggctctctagtgtcccgggaagccagcgataaggaaaaggttaaagaactgccaacact<br>aaaggatatggactttcttaacaagaaccagaaagtgtatattggtgaagaagaaaagaaagtattcctgg<br>agaagctgaagcgagatgtggagtttctagtgcagctgaagatcatggactacagcctcctgttgggcatcc<br>acgacatcatccggggctctgaacccgaggaagaggggcctgtgagggaggaggagtctgagtgggat<br>ggggactgtaacctggctggacctcccgccctggtgggctcctatggtacctcccctgagggtatcggaggc<br>tacatccattcccaccggccactgggcccaggagagtttgagtccttcatcgatgtctatgctatccggagtgc<br>ggagggggcccccagaaggaggtgtatttcatgggcctcattgacattctgacacagtatgatgccaaga<br>agaaagcagctcatgcagccaagactgtcaagcacggggcgggggcagagatctccactgtccatcctg<br>agcagtatgctaagcgattcctggactttattgccaacatctttgcctaa
Mutant-<br>Pip4k2c	atggcgtcctcctccgtccctcccgccaccgcacccgcggcagctggaggccccggcccgggattcggctt<br>cgcctccaaaaccaagaagaagcatttcgtacagcagaaagtgaaggtgttccgggccgcggacccgct

	ggtgggcgtgttcctgtggggcgtcgctcactcgatcaatgagctcagccaggtaccgcccccagaaatttt ccttcctaataattttaaagccagctccaagatcaaggtcaacaatcactttttccatagagaaaatcttccca gtcatttcaagttcaaggagtattgtccccaggtcttcaggaacctgagagatcggtttgccatcgatgatcac gattacttggtgtcccttactcgaagcccccccaagcgaaactgaaggcagtgatggccgtttccttatctccta tgaccgcactctggtcatcaaggaagtgtcgagcgaagatattgcggatatgcacagcaacctctccaact accaccagtacatagtgaaatgtcatggcaacacgcttctgccccagttcctgggcatgtaccgagtcagtg tagaaaatgaagacagctacatgctcgtgatgcgcaatatgtttagtcatcgtcttcctgtgcataggaagtat gacctcaagggctctctagtgtcccgggaagccagcgataaggaaaaggttaaagaactgccaacacta aaggatatggactttcttaacaagaaccagaaagtgtatattggtgaagaagaaaagaaagtattcctgga gaagctgaagcgagatgtggagtttctagtgcagctgaagatcatggactacagcctcctgttgggcatcca cgacatcatccggggctctgaacccgaggaagaggggcctgtgagggaggaggagtctgagtgggatg gggactgtaacctggctggacctcccgccctggtgggctcctatggtacctcccctgagggtatcggaggct acatccattcccaccggccactgggcccaggagagtttgagtccttcatcgatgtctatgctatccggagtgc ggagggggcccccccagaaggaggtgtatttcatgggcctcattgacattctgacacagtatgatgccaaga agaaagcagctcatgcagccaagactgtcaagcacggggcgggggcagagatctccactgtccatcctg agcagtatgctaagcgattcctggactttattgccaacatctttgcctaa
Luc	atggccgatgctaagaacattaagaagggccctgctcccttctaccctctggaggatggcaccgctggcga gcagctgcacaaggccatgaagaggtatgccctggtgcctggcaccattgccttcaccgatgcccacattg aggtggacatcacctatgccgagtacttcgagatgtctgtgcgcctggccgaggccatgaagaggtacggc ctgaacaccaaccaccgcatcgtggtgtgctctgagaactctctgcagttcttcatgccagtgctgggcgccc tgttcatcggagtggccgtggcccctgctaacgacatttacaacgagcgcgagctgctgaacagcatgggc atttctcagcctaccgtggtgttcgtgtctaagaagggcctgcagaagatcctgaacgtgcagaagaagctg cctatcatccagaagatcatcatcatggactctaagaccgactaccagggcttccagagcatgtacacattc

	gtgacatctcatctgcctcctggcttcaacgagtacgacttcgtgccagagtctttcgacagggacaaaacc<br>attgccctgatcatgaacagctctgggtctaccggcctgcctaagggcgtggccctgcctcatcgcaccgcc<br>tgtgtgcgcttctctcacgcccgcgaccctattttcggcaaccagatcatccccgacaccgctattctgagcgt<br>ggtgccattccaccacggcttcggcatgttcaccaccctgggctacctgatttgcggctttcgggtggtgctgat<br>gtaccgcttcgaggaggagctgttcctgcgcagcctgcaagactacaaaattcagtctgccctgctggtgcc<br>aaccctgttcagcttcttcgctaagagcaccctgatcgacaagtacgacctgtctaacctgcacgagattgcc<br>tctggcggcgccccactgtctaaggaggtgggcgaagccgtggccaagcgctttcatctgccaggcatccg<br>ccagggctacggcctgaccgagacaaccagcgccattctgattaccccagagggcgacgacaagcctg<br>gcgccgtgggcaaggtggtgccattcttcgaggccaaggtggtggacctggacaccggcaagaccctgg<br>gagtgaaccagcgcggcgagctgtgtgtgcgcggccctatgattatgtccggctacgtgaataaccctgag<br>gccacaaacgccctgatcgacaaggacggctggctgcactctggcgacattgcctactgggacgaggac<br>gagcacttcttcatcgtggaccgcctgaagtctctgatcaagtacaagggctaccaggtggccccagccga<br>gctggagtctatcctgctgcagcaccctaacattttcgacgccggagtggccggcctgcccgacgacgatg<br>ccggcgagctgcctgccgccgtcgtcgtgctggaacacggcaagaccatgaccgagaaggagatcgtg<br>gactatgtggccagccaggtgacaaccgccaagaagctgcgcggcggagtggtgttcgtggacgaggtg<br>cccaagggcctgaccggcaagctggacgcccgcaagatccgcgagatcctgatcaaggctaagaaag<br>gcggcaagatcgccgtgtaa

Table S2: Antibodies used in this study (FACS)

Antigen	Dilution	Company	Catalog number
CD19 PerCP-Cy5.5	1:300	BioLegend	Clone 6D5
CD49b (pan-NK cells) PerCP-Cy5.5	1:300	BioLegend	Clone DX5
TER-119/Erythroid Cells PerCP-Cy5.5	1:300	BioLegend	Clone TER-119
CD11b APC	1:300	BioLegend	Clone M1/70
CD45 Alexa Fluor 700	1:300	BioLegend	Clone 30-F11
F4/80 PE-Cy7	1:300	BioLegend	Clone BM8
Ly-6C BV510	1:300	BioLegend	Clone HK1.4
Ly-6G FITC	1:300	BioLegend	Clone 1A8
CD3 BV570	1:300	BioLegend	Clone 17A2
CD4 PE-Cy5	1:300	BioLegend	Clone RM4-5
CD8a BV650	1:300	BioLegend	Clone 53-6.7
CD19 PerCP-Cy5.5	1:300	BioLegend	Clone 6D5

Table S3: Antibodies used in this study (immunostaining and western blot)

Antigen	Dilution	Company	Catalog number
α-Actinin	1:200	Abcam	ab946
Pip4k2c	1:100	Proteintech	17077-1-AP
Pip4k2c	1:1000	MyBioSource	MBS9203628
Pip5k1α	1:1000	Novus	NBP2-19833
phospho-AKT 1/2/3	1:1000	Abcam	ab192623
phospho-Smad3 (208)	1:1000	Abcam	ab138659
pH3	1:200	Millipore	06-570
Troponin I	1:50	Santa Cruz Biotechnology	SC-15368
CD45	1:100	BD Pharmingen	550539
Phospho-p70 S6 Kinase (Thr389)	1:2000	Cell Signaling	9205
GFP	1:500	Abcam	ab13970
p70 S6 Kinase	1:2000	Cell Signaling	9202
WGA	1:50	Life Technology	W11261
Vimentin	1:200	Abcam	Ab24525
Troponin I	1:200	Abcam	Ab47003
β-actin	1:2000	Cell Signaling	12262
GAPDH	1:1000	Cell Signaling	8884

Table S4: Primer Sequences for qPCR in this study

Gene	Forward	Reverse
mPip4k2c	ggagactatggcgtcctcct	ccggaacaccttcactttct
18s	agtccctgccctttgtacaca	cgatccgagggcctcacta
FN1	aggaagccgaggttttaactg	aggacgctcataagtgtcacc
Col1a1	ctggcaagaagggagatga	caccatccaaaccactgaaa
Col1a2	aggtcttcctggagctgatg	ccccacagggccttctttac
hPip4k2c	gtggggtagagagtgggtca	tgctgtgcccagatgtagag
Col3a1	acagcaaattcacttacacagttc	ctcattgccttgcgtgttt
ctgf1	aggaagccgaggttttaactg	aggaagccgaggttttaactg
MYHC	cagaacaccagcctcatcaa	gctccttcttcagctcctca
ANP	gcttccaggcatattggag	gggggcatgacctcatctt
BNP	aagggtctggctgctttg	cagccaggacttcctcttaatg
mmp-2	aggacgctcataagtgtcacc	aggaagccgaggttttaactg
mmp-3	gctgccatttctaataaaga	gcacttcctttcacaaag
mmp-7	ttgaaggatggcaagtatgg	cgaaggcatgacctagagtgt
mmp-9	aaggacggccttctggcacacgccttt	gtggtatagtgggacacatagtgg
mt1-mmp	gagatcaaggccaatgttcggagg	cggtagtacttattgcccaag
TGF-β	cctgtccaaactaaggc	ggttttctcatagatggcg

3) Supplemental Figures

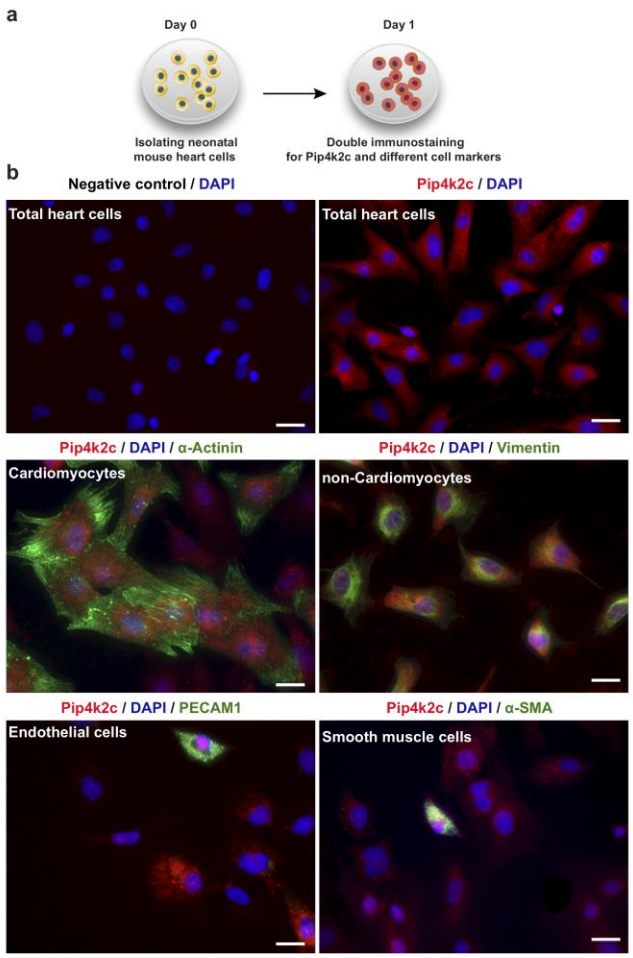

Figure S1. Heart cells, including CM and non-CM (cardiac fibroblasts, endothelial, and smooth muscle cells) express Pip4k2c *in vitro*.

a. Experimental plan to evaluate Pip4k2c expression in isolated, cultured neonatal mouse (P8) heart cells. **b.** Representative images of isolated, cultured neonatal mouse (P8) heart cells stained for Pip4k2c (red); α-Actinin (CM marker, green); Vimentin (non-CM marker, green); PECAM1 (endothelial cell marker, green); α-smooth muscle actin (smooth muscle cell marker, green); DAPI (nuclei marker, blue). Scale bar = 10μm.

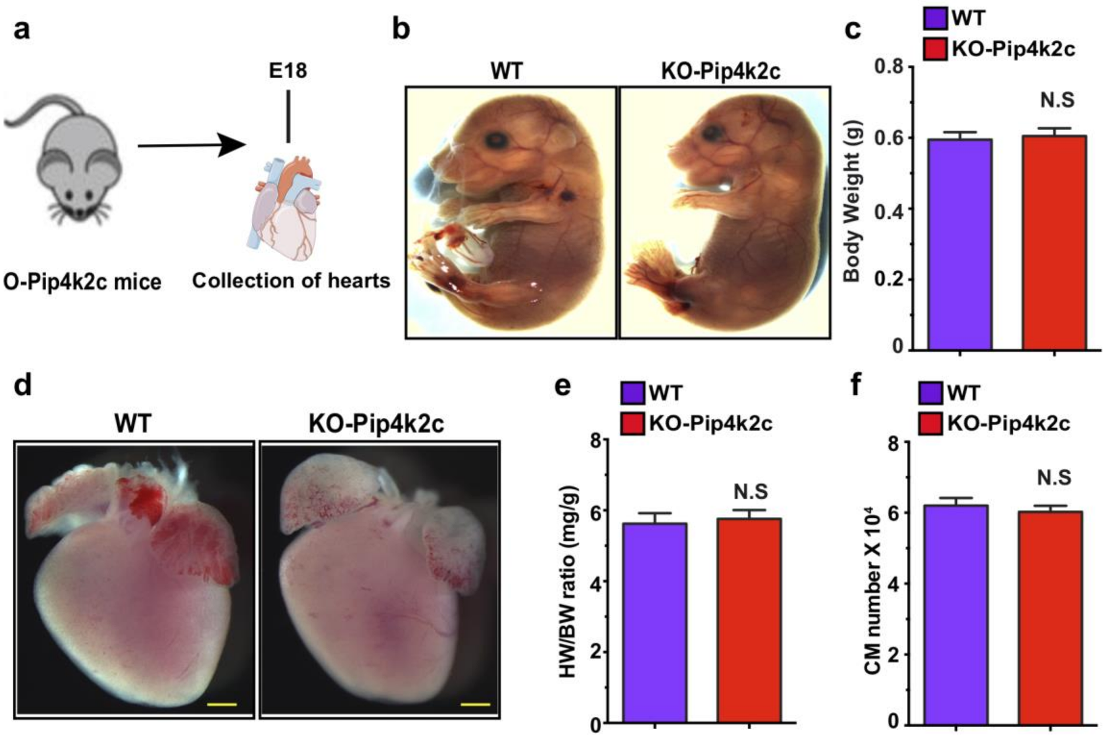

Figure S2. Loss of Pip4k2c does not significantly affect mouse heart development.

a. Experimental plan for studying mouse heart development in Pip4k2c$^{+/+}$ littermate control (WT) or Pip4k2c$^{-/-}$ (KO-Pip4k2c) E18 mice. **b&c.** Representative images (b) and body weight measurements (c) of E18 WT or KO-Pip4k2c mice (n=5). **d.** Representative images of the whole heart. **e&f.** Heart weight to body weight ratio (e, n=5) and quantification of isolated CM (f) from the hearts of E18 WT or KO-Pip4k2c mice (n=3). Unpaired two-tailed t-test. N.S., Not Significant. Scale bar = 1mm (d).

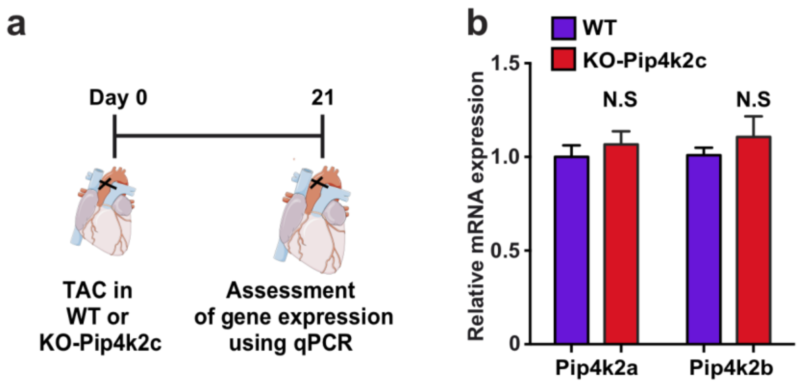

Figure S3. Loss of Pip4k2c has no significant effect on Pip4a2a and Pip4k2c expression post TAC injury.

a. Experimental timeline to evaluate Pip4a2a and Pip4k2b expression in Pip4k2c$^{+/+}$ littermate control (WT) or Pip4k2c$^{-/-}$ (KO-Pip4k2c) in a TAC mouse model. **b.** qPCR analysis of Pip4a2a and Pip4k2b expression 21 days post TAC injury in WT or KO-Pip4k2c (n=5). Unpaired two-tailed t-test for b. N.S., Not Significant.

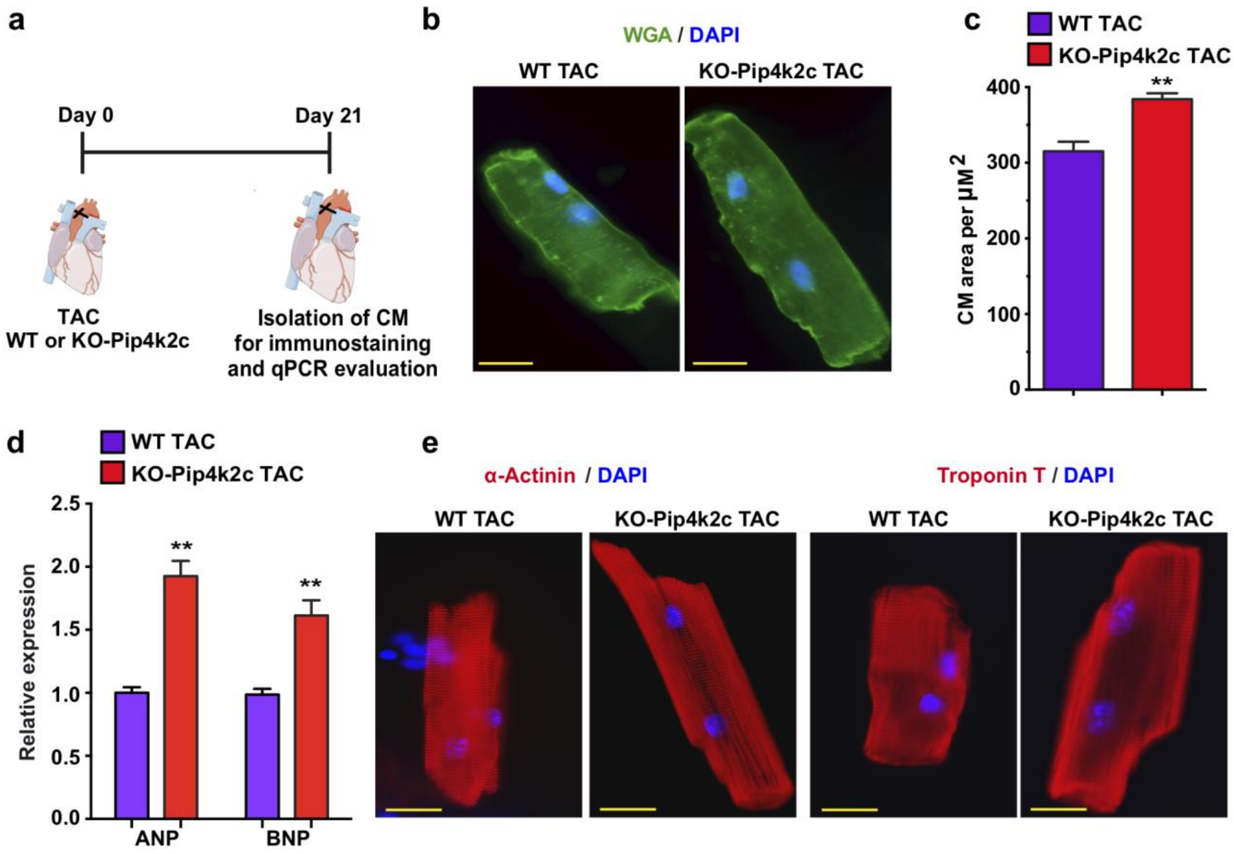

Figure S4. Loss of Pip4k2c induces cardiac hypertrophy.

a. Experimental plan to isolate CM from hearts of Pip4k2c$^{+/+}$ littermate controls (WT) or Pip4k2c$^{-/-}$ (KO-Pip4k2c) mice 21 days post TAC injury. **b.** Representative images of CM isolated from the hearts of WT or KO-Pip4k2c mice 21 days post TAC injury and immunostained with WGA to evaluate CM size (cross-sectional area). **c.** Quantitative analysis of b (n=3). **d.** qPCR analysis of hypertrophic markers in CM isolated from hearts of WT or KO-Pip4k2c mice 21 days post TAC injury (n=3). **e.** Representative images of CM isolated from the hearts of WT or KO-Pip4k2c mice 21 days post TAC injury and immunostained with CM sarcomeric structural proteins as α-Actinin or Troponin T. Unpaired two-tailed t-test, **, P<0.01. Scale bar = 20 μm.

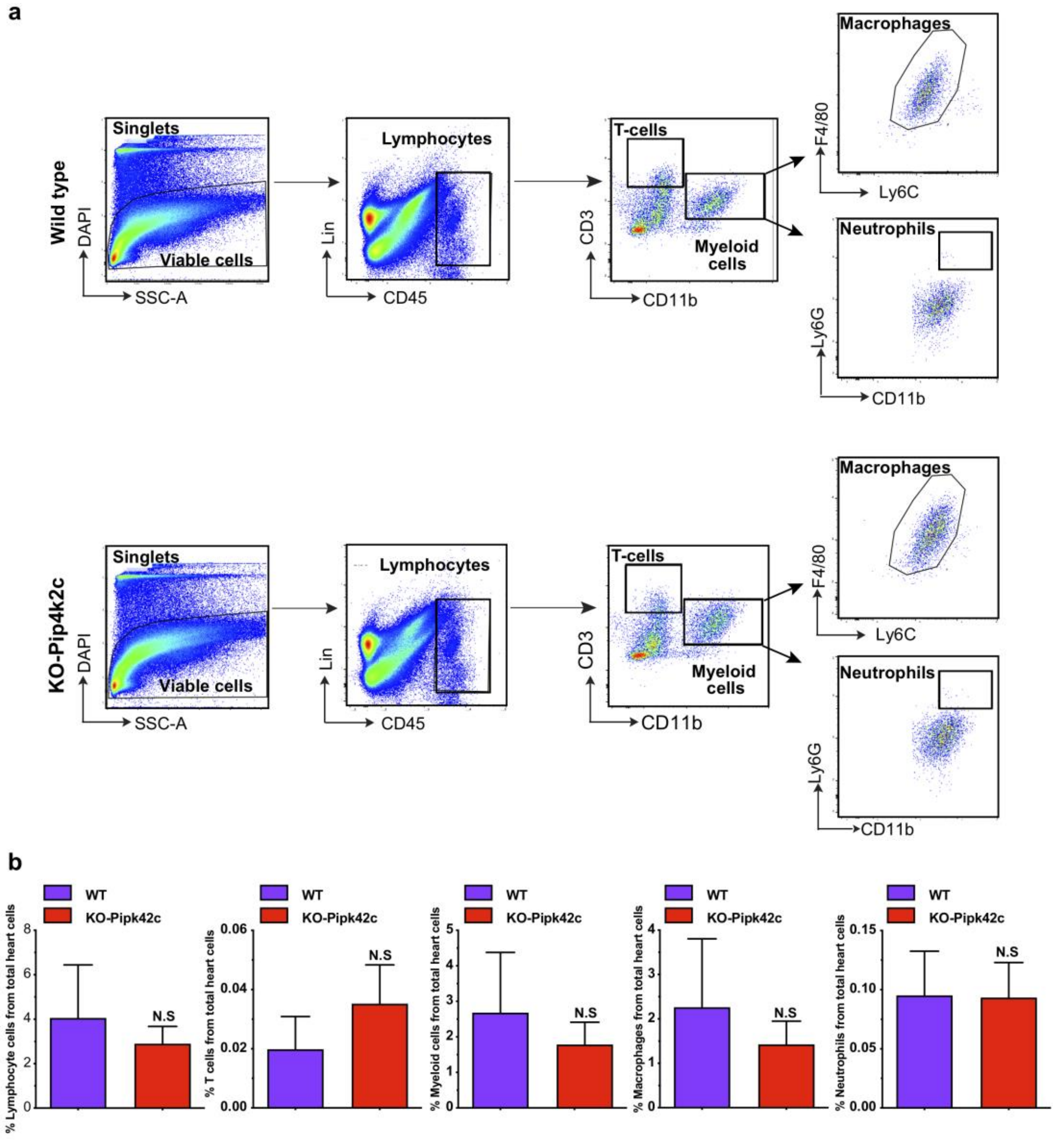

Figure S5. Pip4k2c depletion does not alter immune cell composition in the heart 21 days post TAC injury.

a. Gating strategy for immune cell populations in KO-Pip4k2c versus wild type (WT) hearts 21 days post TAC injury. Cell populations were defined as follows: lymphocytes as Lin+ and CD45+ cells, T-cells as CD3+ lymphocytes, myeloid cells as CD11b+ lymphocytes, macrophages as F4/80+ and Ly6Clow/interm myeloid cells, and neutrophils as Ly6Ghigh and CD11bhigh. **b.** Flow cytometric quantification of a (n=5). Two-tailed Student’s t-tests (b). N.S., Not significant.

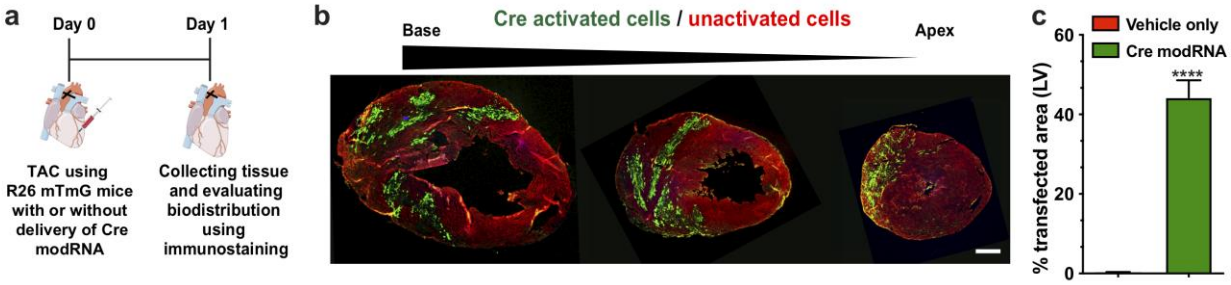

<u>Figure S6. Biodistribution of modRNA transfection in TAC mouse model.</u>

a. Experimental timeline to evaluate modRNA biodistribution in the TAC mouse model. **b.** Representative images of a transfected cross-sectioned heart (short axis view; transfected cells are green, and non-transfected cells are red) in Rosa26mTmG mice 24 hours after 100μg Cre modRNA was injected directly into the myocardium. **c.** Quantification of Cre modRNA biodistribution in the LV post transfection *in vivo* (n=3). Two-tailed Student's t-tests (b). ****, P<0.0001. Scale bar = 1mm.

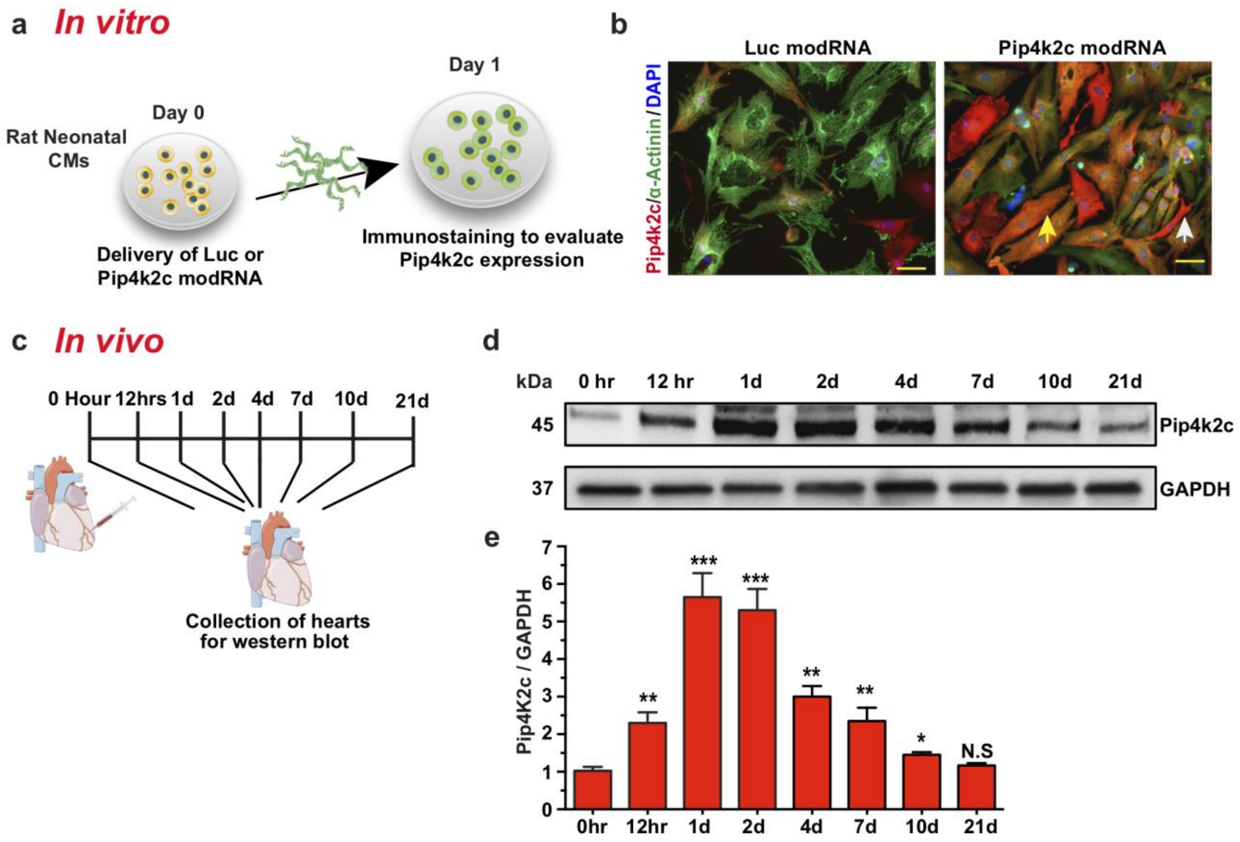

<u>Figure S7. Pip4k2c modRNA expression *in vitro* and pharmacokinetics *in vivo.*</u>

a. Experimental plan to determine Pip4k2c overexpression in P3 neonatal rat CM *in vitro*. **b.** Representative images of the Pip4k2c (red) expression in P3 neonatal rat CM after delivery of Luc (control) or Pip4k2c modRNA, co-stained with α-actinin (CM-marker, green) and DAPI (nuclei, blue). White arrowheads point to non-CM. Yellow arrowheads point to CM. **c.** Experimental plan for evaluating Pip4k2c modRNA pharmacokinetics *in vivo*. **d.** Western blot analysis of Pip4k2c expression post modRNA delivery *in vivo* (n=2). **e.** Quantitative analysis of d (n=2). One-way ANOVA, Tukey's Multiple Comparison Test were used in e. ***, P<0.001, **, P<0.01, *, P<0.05. N.S., Not Significant. Scale bar = 50μm.

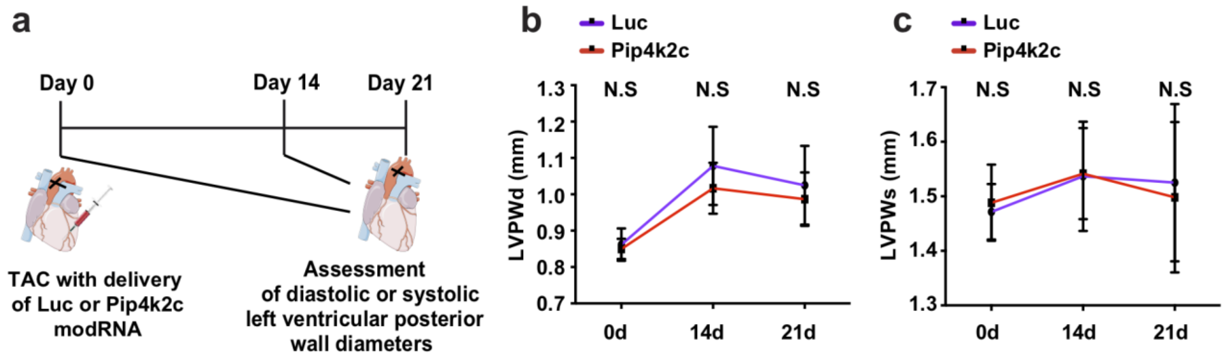

<u>Figure S8. Pip4k2c increase, induced using Pip4k2c modRNA, does not change diastolic or systolic left ventricular posterior wall diameter post TAC injury.</u>

a. Experimental timeline to evaluate Pip4k2c modRNA's effect on diastolic (LVPWd) or systolic (LVPWs) left ventricular posterior wall diameter in a TAC mouse model. **b&c.** Quantitative analysis of LVPWd (b) and LVPWs (c). n=6. Two-way ANOVA, N.S., Not Significant.

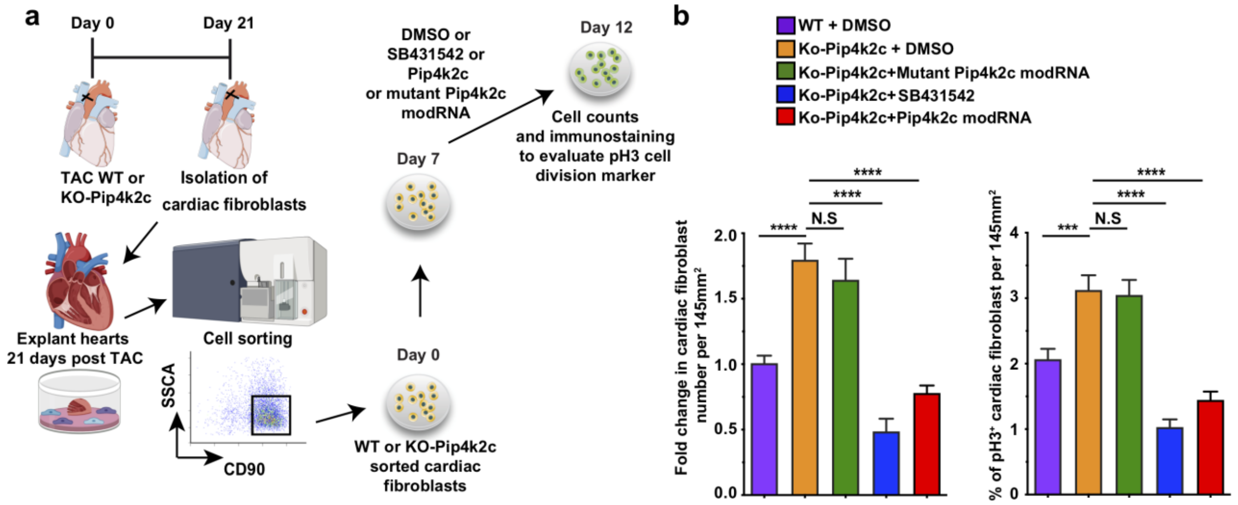

Figure S9. Loss of Pip4k2c induces cardiac fibroblast proliferation, while Pip4k2c (but not mutant Pip4k2c) gains via modRNA inhibit cardiac fibroblast proliferation in isolated and sorted cardiac fibroblasts 21 days post TAC injury.

a. Experimental plan to evaluate Pip4k2c's impact on cardiac fibroblast proliferation. Cardiac fibroblasts were isolated and sorted (for the fibroblastic marker CD90) from WT or KO-Pip4k2c hearts 21 days post TAC injury and treated with DMSO (control), TbetaR1/ALK5 inhibitor (SB431542), Pip4k2c, or mutant Pip4k2c modRNA. Cell numbers and pH3 (cell division marker) were calculated 5 days later. **b.** Quantitative analysis of cell numbers or pH3-positive cardiac fibroblasts in each treatment group (n=3). One-way ANOVA, ****, P<0.0001, ***, P<0.001, **, P<0.01, N.S., Not Significant.

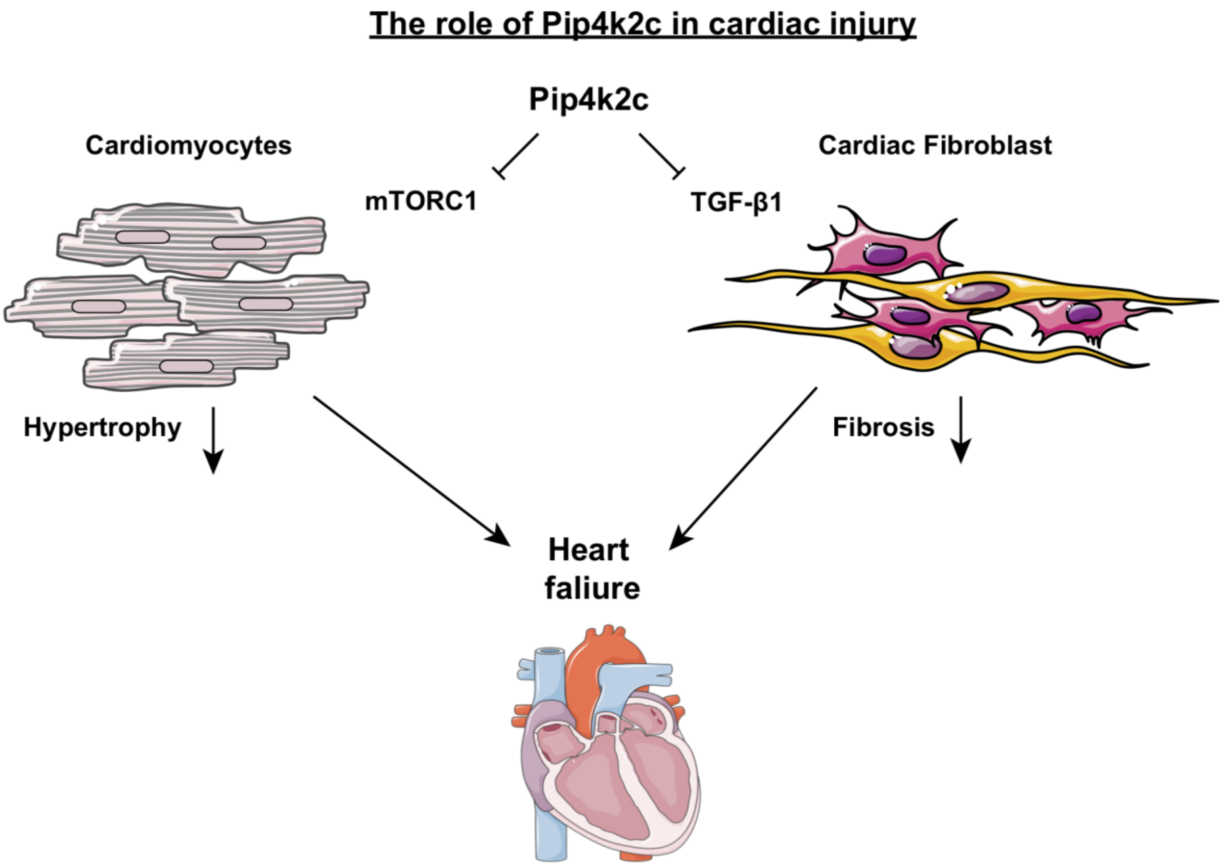

Figure S10. The molecular pathway regulated by Pip4k2c in a TAC mouse model.

4. Author Contributions

A.M. designed and carried out most of the experiments, analyzed most of the data, and wrote the manuscript. N.S. performed experiments and analyzed qRT-PCR and immunostaining data. A.K. performed histology staining, immunostaining, and western blot. M.T.K.S. prepared modRNAs. N.S. performed immunostaining. E.C. performed all mouse surgery and Echo analyses. K.K. sorted and cultured cardiac fibroblasts. M.M.Z. performed FACS analysis of KO-Pip4k2c and WT mice. Y.H. analyzed qRT-PCR data. D.L. provided human heart samples. S.S. and R.J.H. revised the manuscript. L.Z. designed experiments, analyzed data, and wrote the manuscript.
